# Aggregation-induced barrier to oxygen—a new AIE mechanism for metal clusters with phosphorescence

**DOI:** 10.1093/nsr/nwab216

**Published:** 2021-11-30

**Authors:** Yan Jin, Qiu-Chen Peng, Si Li, Hui-Fang Su, Peng Luo, Ming Yang, Xin Zhang, Kai Li, Shuang-Quan Zang, Ben Zhong Tang, Thomas C W Mak

**Affiliations:** Henan Key Laboratory of Crystalline Molecular Functional Materials, Henan International Joint Laboratory of Tumor Theranostical Cluster Materials, Green Catalysis Center, and College of Chemistry, Zhengzhou University, Zhengzhou 450001, China; Henan Key Laboratory of Crystalline Molecular Functional Materials, Henan International Joint Laboratory of Tumor Theranostical Cluster Materials, Green Catalysis Center, and College of Chemistry, Zhengzhou University, Zhengzhou 450001, China; Henan Key Laboratory of Crystalline Molecular Functional Materials, Henan International Joint Laboratory of Tumor Theranostical Cluster Materials, Green Catalysis Center, and College of Chemistry, Zhengzhou University, Zhengzhou 450001, China; Henan Key Laboratory of Crystalline Molecular Functional Materials, Henan International Joint Laboratory of Tumor Theranostical Cluster Materials, Green Catalysis Center, and College of Chemistry, Zhengzhou University, Zhengzhou 450001, China; Henan Key Laboratory of Crystalline Molecular Functional Materials, Henan International Joint Laboratory of Tumor Theranostical Cluster Materials, Green Catalysis Center, and College of Chemistry, Zhengzhou University, Zhengzhou 450001, China; Henan Key Laboratory of Crystalline Molecular Functional Materials, Henan International Joint Laboratory of Tumor Theranostical Cluster Materials, Green Catalysis Center, and College of Chemistry, Zhengzhou University, Zhengzhou 450001, China; Henan Key Laboratory of Crystalline Molecular Functional Materials, Henan International Joint Laboratory of Tumor Theranostical Cluster Materials, Green Catalysis Center, and College of Chemistry, Zhengzhou University, Zhengzhou 450001, China; Henan Key Laboratory of Crystalline Molecular Functional Materials, Henan International Joint Laboratory of Tumor Theranostical Cluster Materials, Green Catalysis Center, and College of Chemistry, Zhengzhou University, Zhengzhou 450001, China; Henan Key Laboratory of Crystalline Molecular Functional Materials, Henan International Joint Laboratory of Tumor Theranostical Cluster Materials, Green Catalysis Center, and College of Chemistry, Zhengzhou University, Zhengzhou 450001, China; Shenzhen Institute of Aggregate Science and Technology, School of Science and Engineering, Chinese University of Hong Kong, Shenzhen 518172, China; The Hong Kong Branch of the Chinese National Engineering Research Center for Tissue Restoration and Reconstruction, Hong Kong University of Science and Technology, HongKong, China; Henan Key Laboratory of Crystalline Molecular Functional Materials, Henan International Joint Laboratory of Tumor Theranostical Cluster Materials, Green Catalysis Center, and College of Chemistry, Zhengzhou University, Zhengzhou 450001, China; Department of Chemistry, Chinese University of Hong Kong, Hong Kong, China

**Keywords:** metal clusters, aggregation-induced emission, phosphorescence, photoresponse, O_2_ detection

## Abstract

Metal clusters are useful phosphors, but highly luminescent examples are quite rare. Usually, the phosphorescence of metal clusters is hindered by ambient O_2_ molecules. Transforming this disadvantage into an advantage for meaningful applications of metal clusters presents a formidable challenge. In this work, we used ligand engineering to judiciously prepare colour-tuneable and brightly emitting Cu(I) clusters that are ultrasensitive to O_2_ upon dispersion in a fluid solution or in a solid matrix. When the O_2_ scavenger dimethyl sulfoxide (DMSO) was used as the solvent, joint photo- and oxygen-controlled multicolour switches were achieved for the first time for metal cluster-based photopatterning and photo-anticounterfeiting. More importantly, an aggregation-induced barrier to oxygen, a new aggregation-induced emission mechanism for metal clusters, was proposed, providing a new pathway to realizing the intense emission of metal clusters in the aggregated state. These results are expected to promote the application of metal clusters and enrich the luminescence theory of metal cluster aggregates.

## INTRODUCTION

Metal clusters consist of multiple metal atoms bonded by metal–metal interactions and protective organic ligands [1,2]. These clusters have intermediate sizes, between those of atoms and nanoparticles, and unique geometries and electronic structures, enabling absorption/emission over a wide wavelength range from UV to NIR, which opens up their multiple application prospects in the development of luminescent materials [3–5]. However, due to the participation of metal moieties, the emission of metal clusters is typically classified as long-lived phosphorescence, with lifetimes generally >10 μs [6–8]. Thus, ambient O_2_ molecules inevitably consume the energy of the triplet states and block the luminescence. To develop practical luminescent applications of emissive metal clusters, stable cluster materials with high inherent emission must first be designed and prepared; moreover, the negative effects of O_2_ should be avoided, or this disadvantage should be transformed into an advantage.

Aggregation-induced emission (AIE), a term coined by B.Z. Tang, has been used to explain various issues in which luminogens are dark in the aggregated state [9–11]. The ongoing development of luminescent materials has also promoted the elucidation of AIE mechanisms, such as restriction of intramolecular rotation (RIR), restriction of intramolecular vibration (RIV), excited-state intramolecular proton transfer (ESIPT) and clusterization-triggered emission (CTE) [12–16]. After approximately two decades of development, types of AIE molecules have increased, from small organic molecules to polymers, natural macromolecules, complexes and metal clusters [17–22]. The emission performance of metal clusters, a newly emerging type of emitter, in the aggregate state may benefit future technological applications and contribute to understanding the roles of intracluster chemical events during emission. To date, some excellent cases of AIE metal clusters that follow the above AIE mechanisms have been reported [23–28]. However, it is unknown whether there are other mechanisms of emission or emission enhancement for aggregates or crystalline metal clusters.

In this work, a series of trinuclear copper clusters was prepared. These clusters exhibited intense phosphorescence with different emission wavelengths. The emission of each metal cluster was ultrasensitive to O_2_ when the clusters were dispersed in a fluid solution or in a solid matrix. Thus, we developed a multicoloured visualized photochemical process by jointly using UV light irradiation and a deoxygenating solvent to assemble prototypes of photopatterning and anti-counterfeiting codes, thereby extending the functional applications of isolated metal clusters. More importantly, an aggregation-induced barrier to oxygen (AIBO), a new AIE mechanism for metal clusters, is proposed. In the dispersed state, phosphorescence of the clusters is quenched by O_2_ in solvents, whereas in the aggregated state, the molecules on the surface of the aggregates form a barrier, preventing direct contact between O_2_ and the cluster molecules; thus, intense phosphorescence can be observed. The AIBO mechanism provides a new pathway for the intense emission of metal clusters in the aggregated state. Such intense emission endows metal clusters with a cell imaging capability that has a high co-localization coefficient, making the metal clusters suitable for constructing light-emitting devices.

## RESULTS AND DISCUSSION

### Synthesis and structure of the copper clusters

**Figure 1.**
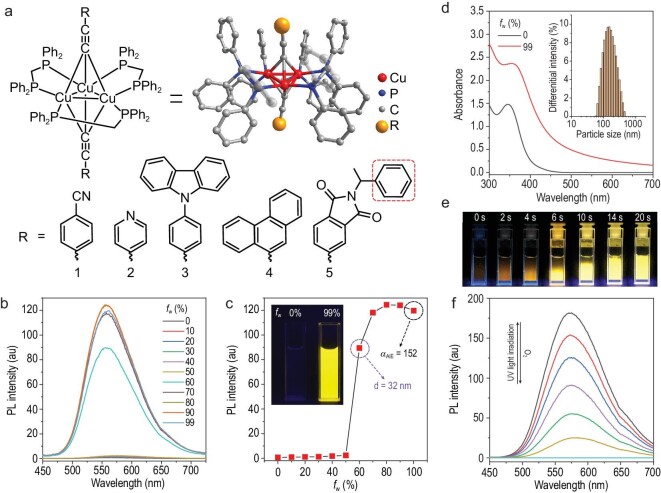
Structures and luminescence properties of the clusters. (a) The structures of clusters **1**–**5**. (b) Luminescence spectra of cluster **1** in H_2_O/EtOH mixtures with different *f*_w_ values. (c) Luminescence intensity of cluster **1** at 559 nm as a function of *f*_w_. Inset: photograph of cluster **1** in 0% and 99% H_2_O/EtOH mixtures. (d) Absorption spectra of cluster **1** in H_2_O/EtOH mixtures with different *f*_w_ values. Inset: DLS results of cluster **1** in a 99% H_2_O/EtOH mixture. (e) Photographs of cluster **1** in dimethyl sulfoxide (DMSO) upon UV light irradiation. (f) Luminescence spectra of cluster **1** in DMSO upon UV light irradiation. Condition: [**1**] = 50 μmol/L.

Trinuclear copper clusters were prepared using a precursor of [Cu_2_(μ-dppm)_2_(MeCN)_2_][PF_6_]_2_ (dppm is bis(diphenylphosphino)-methane) through a solvent diffusion method [29,30]. The precursor and corresponding alkyne ligands were dissolved in THF with excess KOH. The mixture was filtered after stirring for 24 h at room temperature. The filtrate was diffused with diethyl ether to form crystals. The structures of the crystals were determined by single-crystal X-ray analysis, and the phase purity of the clusters was confirmed by powder X-ray diffraction (Supplementary Fig. S5). As shown in Fig. 1a and Supplementary Figs S6 and S7, three copper atoms formed a regular triangle kernel, and each dppm ligand connected two copper atoms to form a side of the triangle, leading to a Cu_3_P_6_ core. The methylene carbon atoms of the dppm ligand were located on the flap of each Cu_2_P_2_C ring. Furthermore, two alkynyl ligands were each C-bonded to three copper atoms in the *μ*_3_-*η*^1^ bridging modes. Notably, the alkyne triple bond axes were not perfectly perpendicular to the Cu_3_ plane but slanted slightly towards the Cu–C edge with the longest Cu–C bond distances. The C≡C–Cu bond angles between the acetylide group and each of the copper atoms ranged from 111.6(3)° to 159.6(2)°. The alkyne triple bond lengths ranged from 1.107(16) Å to 1.27(4) Å, indicating metal acetylide σ bonding [31,32]. In addition, the distance between adjacent copper atoms ranged from 2.4642(5) Å to 2.788(2) Å, a distance that is shorter than the sum of van der Waals radii of copper (2.8 Å) [33], demonstrating the presence of metal–metal interactions. The only exception was cluster **3**, in which the distance between Cu1 and Cu3 was 3.263 Å. This distance is too long to form Cu–Cu bonds and led to a more distorted Cu_3_P_6_ core (Supplementary Tables S8–S17).

### Luminescence properties of the copper clusters

Different alkynyl ligands endowed the metal cluster crystals with different intense luminescence properties (Table 1). The lifetimes of the metal clusters were determined to range from 26 μs to 59 μs, demonstrating their phosphorescence properties. According to the literature, the phosphorescence of these metal clusters originates from a metal-to-ligand charge transfer (MLCT) process, which is favourable for spin-orbit coupling in the clusters [29]. Interestingly, when the metal clusters were dissolved in EtOH, no emission was observed, suggesting that they have AIE characteristics [34]. Thus, the emission spectra of the metal clusters in EtOH/H_2_O mixtures with different water fractions (*f*_w_) were investigated. Taking cluster **1** as an example, an intense emission was observed in water-dominated solutions, and the wavelength was highly similar to that of the emission observed in the crystalline state. With an increase in *f*_w_, the emission intensity increased gradually because aggregates formed (Fig. 1b and c). The presence of these aggregates was confirmed by absorption spectroscopy and dynamic light scattering (DLS) measurements. As shown in Fig. 1d, a level-off tail was clearly observed in the visible region of the absorption spectrum of cluster **1** in a water-dominant solution due to light scattering by the aggregated suspensions [35]. The DLS results showed that particles of ∼200 nm were present in the water-dominated solution, but no particles were present in the EtOH. Particles of ∼30 nm were observed in the water-containing solutions with initial visible emission. In addition, according to the crystal structure, the size of the cluster was ∼2 nm, which means that there might be at least ∼3000 molecules in the aggregate to express emission enhancement (Supplementary Fig. S8). Similarly, AIE characteristics were also observed for metal clusters **2**–**4**, and the data are shown in Supplementary Figs S9–S11. The *α*_AIE_ values of clusters **1**–**4**, which were calculated by *I*/*I*_0_ (*I* is the emission intensity in a 99% H_2_O/EtOH mixture, and *I*_0_ is the emission intensity in EtOH), are shown in Table 1 [36].

**Table 1.** Luminescence lifetime, emission wavelength, absorption wavelength and *α*_AIE_ value of the metal clusters in different states.

	In crystal state			In water-dominated solution				In DMSO after UV light irradiation		
	*~τ* (μs)	*λ*_Em_ (nm)	*λ*_abs_ (nm)	*τ* (μs)	*λ*_Em_ (nm)	*λ*_abs_ (nm)	*α*_AIE_ (%)	t*τ* (μs)	*λ*_Em_ (nm)	*λ*_abs_ (nm)
**1**	36	551	376	18	559	354	152	219	568	348
**2**	26	507	376	22	518	332	164	30	529	327
**3**	40	510	300	31	505	346	68	317	514	343
**4**	59	572	347	8.1	578	356	9.1	542	572	354

To determine the origin of the AIE characteristics of the metal clusters, their luminescence properties in organic solvents with different viscosities were recorded. As shown in Supplementary Fig. S12, the emission intensity of clusters **1**–**4** in the high-viscosity solvent glycerol was much higher than in EtOH, suggesting that their AIE characteristics may originate from an RIR process [37]. As shown in Fig. 1a, many phenyl groups were present in the metal clusters and they underwent active dynamic intramolecular rotation in EtOH, providing a non-radiative transition pathway for the excited-state electrons to return to the ground state. As a result, the luminescence of clusters **1**–**4** in the dissolved state was quenched in EtOH. In glycerol, intramolecular rotation of phenyl groups was effectively restricted by the high-viscosity solvent, and blockage of the non-radiative transition pathway resulted in intense luminescence. As in high-viscosity solvents, aggregates in the solid state can also restrict intramolecular rotation of phenyl groups, allowing the emission of clusters **1**–**4**, i.e. AIE emission.

### Photoresponse of the copper clusters in DMSO

**Figure 2.**
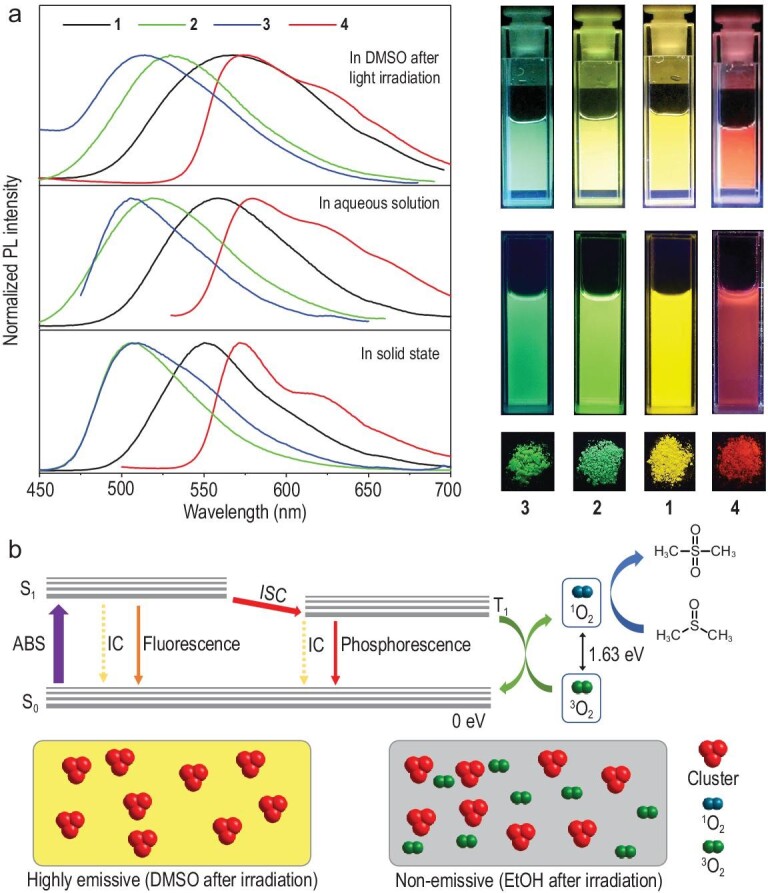
Proposed mechanism for the photoresponse of metal clusters. (a) Luminescence spectra and corresponding luminescence photographs of clusters **1**–**4** in different states. Condition: [**1**–**4**] = 50 μmol/L. (b) A simplified Jablonski diagram to illustrate the production of singlet O_2_ and dimethyl sulfone (top). ABS is absorption, IC is internal conversion and ISC is intersystem crossing. Schematic diagram of the metal cluster-contained solutions after UV light irradiation (down).

Surprisingly, when we irradiated the DMSO stock solution of the metal clusters with UV light at 365 nm, fast luminescence ‘turn-on’ responses were observed (Supplementary Video S1, Fig. 1e and f and Supplementary Fig. S13). Interestingly, the wavelength and peak pattern of these photoinduced turn-on emissions were highly similar to those in the solid state and in water-dominated solutions (Fig. 2a). These results suggest that in addition to the RIR process, a new AIE mechanism may have been involved. Therefore, the photoresponses of clusters **1**–**4** were systematically studied. The photoresponses of the metal clusters were first investigated in different solvents. As shown in Supplementary Fig. S14, after irradiation with UV light (20 mW cm^–2^) for 5 min, only the clusters in DMSO exhibited obvious luminescence enhancement, indicating that the sulfoxide structure in the solvent was necessary for the photoresponse. According to previous reports, O_2_ molecules (^3^Σ_g_) in solvents quench phosphorescence and can be activated by UV light irradiation to form singlet O_2_ (^1^Δ_g_) in the presence of a photosensitizer [38–40]. This singlet O_2_ can then be scavenged by sulfoxide molecules to form sulfone molecules [41–43]. As a result, a photoinduced deoxygenating zone is formed, and the phosphorescent molecules in this zone become emissive. Thus, a possible mechanism for the photoresponse of the metal clusters is proposed (Fig. 2b). The phosphorescence of the metal clusters is quenched by O_2_ molecules without UV light irradiation. Upon UV light irradiation, the O_2_ molecules are converted to singlet O_2_ due to the photosensitivity of the metal clusters. The produced singlet O_2_ reacts with sulfoxide molecules and is depleted, enhancing the phosphorescence of the metal clusters.

**Figure 3.**
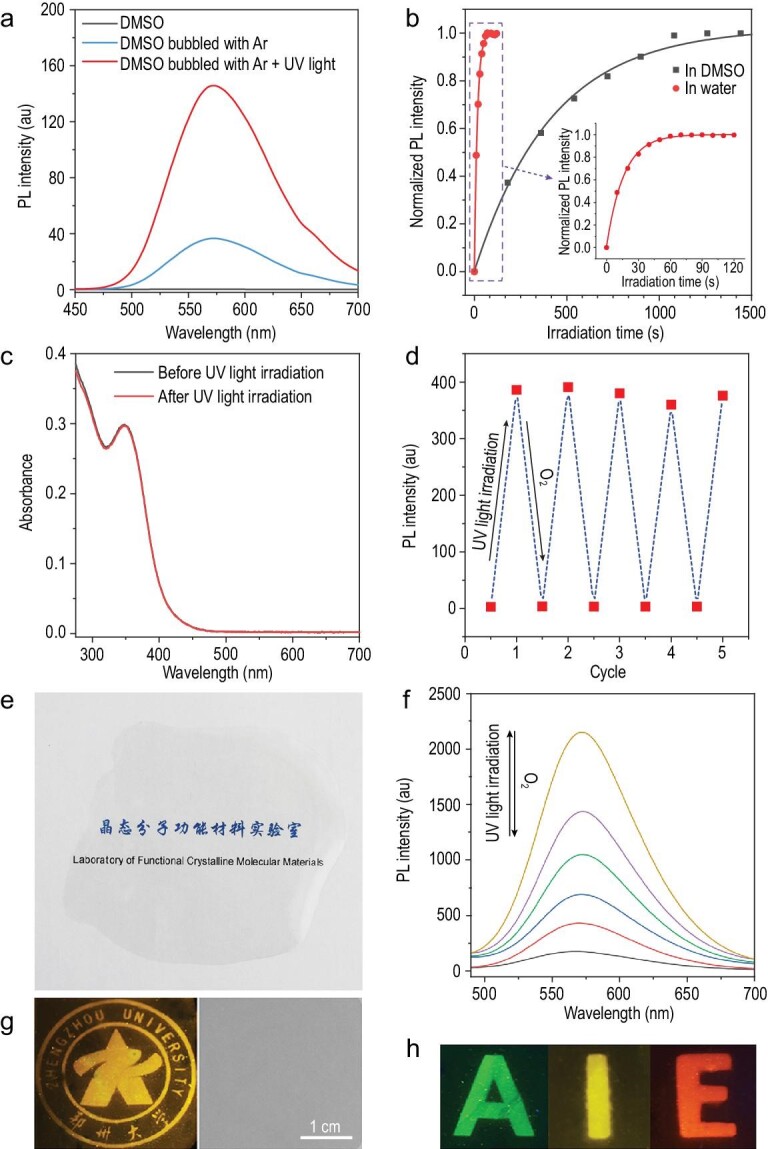
Mechanism study for the photoresponse of metal clusters. (a) Luminescence spectra of cluster **1** in DMSO before and after bubbling with Ar and irradiation with UV light. (b) Fluorescence intensity changes at 525 nm of 5 μmol/L DCFH-DA and 10 μmol/L cluster **1** in an aqueous solution and DMSO upon light irradiation for different times. (c) Absorption spectra of 50 μmol/L cluster **1** in DMSO before and after UV light irradiation. (d) Fatigue resistance of 50 μmol/L cluster **1** upon UV light irradiation and standing in air alternately. (e) Photographs of cluster **1** in PVDF film under daylight. (f) Luminescence spectra of cluster **1** on a PVDF film before and after UV light irradiation. (g) Pattern of the school emblem of Zhengzhou University generated on a PVDF film containing cluster **1** (left: under 365 nm UV light; right: under daylight). (h) Letters ‘AIE’ generated on PVDF films containing clusters **3**, **2** and **4**, respectively.

To verify the mechanism, a series of experiments was carried out. The experimental results for cluster **1** are shown in Fig. 3, and the experimental results for clusters **2**–**4** are provided in the supplementary information (Supplementary Figs S15–S24). O_2_ was removed from the solvents by bubbling Ar into the solvents. As shown in Fig. 3a and Supplementary Fig. S15, the emission of metal clusters increased when Ar was bubbled into the solution. When the system was sealed, the emission remained unchanged for 5 min (Supplementary Fig. S16). In contrast, when air was provided, the emission was quenched immediately. These results demonstrate that the phosphorescence of the metal clusters was quenched by molecular O_2_. Furthermore, the emission intensity of the metal clusters in DMSO bubbled with Ar was weaker than that in DMSO upon UV light irradiation, which was attributed to trace O_2_ present in high-pure Ar (on the order of magnitude of ppm) [44]. In contrast, the O_2_ in DMSO can be removed completely by UV light irradiation in the presence of metal clusters, resulting in a more significant emission enhancement. DCFH-DA was used as an indicator to visualize singlet O_2_ production [45]. As shown in Fig. 3b, the emission of DCFH-DA with cluster **1** in an aqueous solution was rapidly enhanced with irradiation time. The emission intensity of cluster **1** after 60 s of irradiation was 285 times higher than that of the unirradiated sample. In contrast, little emission change was observed for DCFH-DA or cluster **1** alone, demonstrating that cluster **1** is an efficient photosensitizer for singlet O_2_ production. Similar singlet O_2_ production results were also observed for clusters **2**–**4**; the only difference was the time taken to achieve the emission maximum, which ranged from 10 s to 150 s (Supplementary Fig. S17), indicating different singlet O_2_ production rates for the different clusters. Notably, the singlet O_2_ production rate of the metal clusters was positively associated with the emission rate, demonstrating that the emission enhancement of the metal clusters was caused by their photosensitivities. Interestingly, when DCFH-DA and cluster **1** were dissolved in DMSO, the emission enhancement rate of DCFH-DA upon white light irradiation decreased significantly due to the competing reactions of singlet O_2_ between DMSO and cluster **1**. Furthermore, time-dependent density functional theory (TD-DFT) calculations suggested that the energy gaps (Δ*E*_t1-s0_) between the excited triplet states (T_1_) and ground states (S_0_) of the metal clusters were higher than the energy of the excitation from triplet O_2_ to singlet O_2_ (1.63 eV), explaining the origination of the photosensitivity (Fig. 2b) [46]. Interestingly, the energy gaps (Δ*E*_s1-t1_) between excited singlet states (S_1_) and T_1_ of the metal clusters were strongly related to the emission rate upon UV light irradiation. As shown in Supplementary Table S1, a smaller Δ*E*_t1-s0_ led to a shorter phosphorescence time upon UV light irradiation in DMSO. This result demonstrates that electronic transition from S_1_ to T_1_ is the key step in the photoresponse of the metal clusters and that a smaller Δ*E*_s1-t1_ is beneficial for the transfer of excited electrons from S_1_ to T_1_.

^1^H nuclear magnetic resonance (NMR) spectra of cluster **1** in DMSO-*d*_6_ upon UV light irradiation were recorded. As shown in Supplementary Fig. S18, as the irradiation time increased, a peak at 2.96 ppm, attributed to sulfone, emerged and gradually increased, confirming the proposed mechanism. Furthermore, the absorption spectra (Fig. 3c) and high-resolution mass spectra (Supplementary Fig. S20) of cluster **1** in DMSO before and after UV light irradiation were highly similar, demonstrating that cluster **1** remained intact after UV light irradiation. Finally, the luminescence of cluster **1** was reversible by alternating the UV light irradiation and O_2_ exposure for at least five cycles in DMSO, indicating the robust stability of the cluster (Fig. 3d). All these results support the proposed mechanism illustrated in Fig. 2b.

The metal clusters were further dispersed in a porous solid matrix of DMSO-containing PVDF, which was prepared as transparent films (Fig. 3e). The preparation of the PVDF films is described in the supplementary information (for convenience, PVDF film will be used as an abbreviation for the DMSO-containing PVDF film in later discussions). Analogous to those in DMSO, the metal clusters on the PVDF film exhibited very weak luminescence in air. In contrast, intense luminescence was observed from the film after UV light irradiation due to the depletion of O_2_ molecules by DMSO. When the UV light was removed, the PVDF film gradually returned to its original state because the O_2_ molecules in the air came into contact with it (Fig. 3f and Supplementary Fig. S22). The reversible photoresponse of the PVDF film showed excellent fatigue resistance. As shown in Supplementary Fig. S23, when the PVDF films were toggled repeatedly between the luminescence and non-luminescence states 10 times, the maximum luminescence intensity remained almost constant without degradation.

Due to the reversible photoresponse of the PVDF films, the first example of metal cluster-based photopatterning with erasable properties was achieved. As shown in Fig. 3g and h, the letters ‘AIE’ and a pattern of the school emblem of Zhengzhou University were printed on PVDF films containing clusters **1**–**4**, respectively. More interestingly, the information could also be input directly by a 405 nm laser point (Supplementary Video S2). All of the patterns were invisible under daylight, and the absorption spectra of clusters **1**–**4** on the PVDF film before and after UV light irradiation were almost identical (Supplementary Fig. S24). Thus, the film was capable of serving as a material for photo-anticounterfeiting.

### A new AIE mechanism, AIBO, and its applications

**Figure 4.**
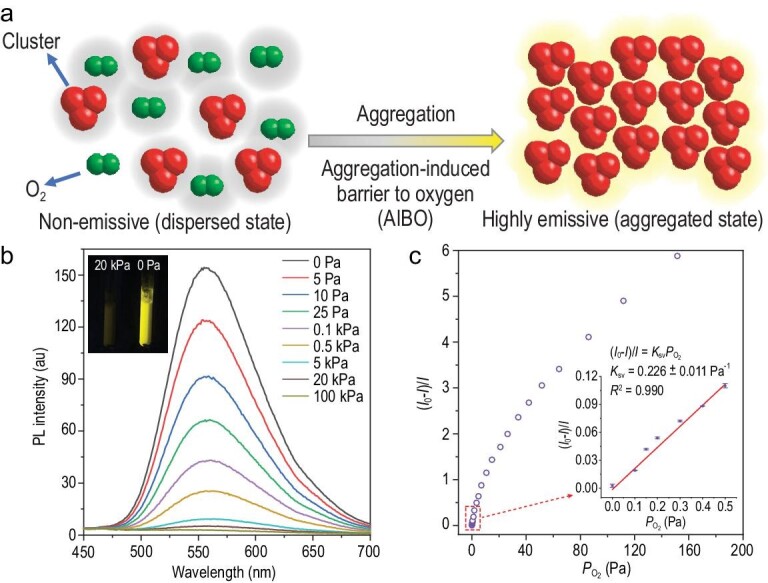
AIBO mechanism for the AIE characteristic of a metal cluster. (a) Schematic diagram of the AIBO process. (b) Emission spectra of cluster **1** in silica gel under different O_2_ pressures from 0 to 100 kPa. Inset: luminescence photograph of cluster **1** in silica gel under O_2_ pressures of 20 kPa and 0 Pa. (c) Correlation between the photoluminescence response and O_2_ partial pressure. Inset: representative Stern-Volmer plot of O_2_ in the range of 0–0.5 Pa. Condition: the mass fraction of cluster **1** in the sample was 0.5%.

Based on the quenching of phosphorescence by O_2_ molecules, AIBO, a new AIE mechanism for phosphorescent metal clusters, was proposed (Fig. 4a). In the dispersed state, the phosphorescence of metal clusters was quenched by adjacent O_2_ molecules dissolved in the solutions. In the aggregated state, the molecules on the surface of the aggregates formed a barrier, preventing the direct contact of triplet O_2_ molecules with the metal clusters and resulting in enhanced emission. To verify the proposed mechanism, cluster **1** was dispersed in a porous solid matrix of silica gel (the mass fraction of cluster **1** was 0.5%). Scanning electron microscopy (SEM) was used to investigate the distribution of **1** on silica gel. Supplementary Fig. S25 shows that the clusters were distributed evenly on the surface of the silica gel. Cluster **1** was dispersed throughout the silica gel, and O_2_ molecules were present throughout the porous solid matrix and effectively quenched the phosphorescence of cluster **1**. As expected, the sample exhibited very weak luminescence in air, although the active dynamic intramolecular rotation of phenyl groups in cluster **1** was restricted (Fig. 4b). In contrast, when the metal cluster in the silica gel was exposed to vacuum, intense luminescence was observed (Supplementary Video S3). These results demonstrate that the AIE properties of phosphorescent metal clusters mainly originate from the AIBO process.

When the metal clusters in silica gel were exposed to alternating air/vacuum, rapid switching between the non-emissive state and the bright-yellow emission state with excellent fatigue resistance was instantaneously observed by visual inspection (Supplementary Video S3). As shown in Supplementary Fig. S26, the luminescent transient response curves of the metal clusters after 30 cycles were recorded without performance loss. More importantly, the luminescence response of cluster **1** in silica gel was ultrasensitive to O_2_, making it suitable for O_2_ detection. As little as 0.1 Pa O_2_ can lead to 1.6% quenching of the phosphorescence of cluster **1** in silica gel. In the range of vacuum (10^–3^ Pa of O_2_) to 0.5 Pa, a good linear Stern-Volmer plot was observed (*R*^2^ = 0.990) (Fig. 4c). The Stern-Volmer constant was calculated to be 0.226 ± 0.011 Pa^–1^, and the limit of detection (LOD) for O_2_ at 1% quenching was 44.2 mPa.

To further understand the contribution of the AIBO process compared with that of the RIR process, the quantum yields (QYs) of clusters **1**–**4** in deoxygenated DMSO (bubbled with Ar for 10 min) and in glycerol were recorded and compared. As shown in Supplementary Table S2, the QYs of clusters **1**–**4** in deoxygenated DMSO were much higher than those in glycerol, indicating that the AIBO process played a more important role in their AIE characteristics. Moreover, control compound **5**, which has two rotatable phenyl groups far from the metal cluster centre compared with those in clusters **1**–**4**, was prepared (Fig. 1a, highlighted with a red box). Similar to clusters **1**–**4**, cluster **5** exhibited typical AIE properties and phosphorescence emission (Supplementary Fig. S27) with a lifetime of 11 μs in the aggregated state, and a remarkable emission of **5** was observed in glycerol (QY = 3%). However, due to the strong dynamic intramolecular rotation of the two phenyl groups, which provided an efficient non-radiative transition pathway for the excited-state electrons to return to the ground state, no emission was observed for cluster **5** in deoxygenated DMSO (QY = ∼0%). These results suggest that only an oxygen-free environment can prevent the emission of **5**. Therefore, both the AIBO process and the RIR process can contribute to the AIE characteristics of phosphorescent metal clusters, and the main influential factors are determined by their structures.

The AIBO process is appropriate for explaining the origin of the AIE property not only of the three core metal clusters but also of other phosphorescent metal clusters. As shown in Supplementary Fig. S28, control compounds **6** and **7**, which have completely different structures than the three core metal clusters, were prepared [47,48]. The luminescence lifetimes of clusters **6** and **7** were 14 μs and 27 μs, respectively. In addition, both clusters **6** and **7** exhibited typical AIE characteristics, with *α*_AIE_ values of 225 and 366, respectively. The phosphorescence of clusters **6** and **7** was also induced by an oxygen-free environment, demonstrating that the AIBO mechanism is a universal mechanism for the origination of AIE properties of phosphorescent metal clusters.

**Figure 5.**
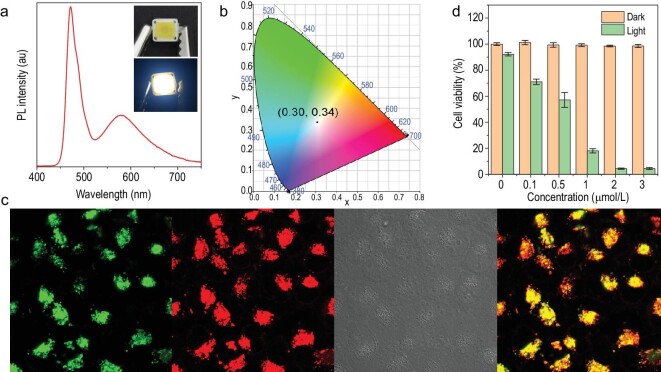
Applications of the metal clusters in aggregated state. (a) Emission spectra and corresponding (b) CIE coordinates of the blue LED chip coated with a film containing cluster **1**. The inset of (a) shows photographs of blue LEDs coated with a film containing cluster **1** when the LED is off (top) and on (bottom). (c) From left to right: co-localization imaging of A549 cells stained with 5 μmol/L **2** (green, 500–650 nm) and 0.1 μmol/L LysoTracker deep red (red, 650–740 nm), bright-field image and merged image of the cells. (d) Viability of the A549 cells stained with different concentrations of cluster **2** in the presence or absence of white light irradiation for 10 min. Light power: 5 mW/cm^2^.

The intense emission of metal clusters in the aggregated state makes them suitable for the construction of lighting devices. As shown in the inset of Fig. 5a, a film containing cluster **1** was added to the surface of a blue LED chip. Upon connection to an electrical power of 2.4 V, the resultant LED device glowed a bright white colour with a CIE coordinate of (0.30, 0.34) (Fig. 5a and b). This result demonstrates that cluster **1** is an ideal yellow phosphor for the construction of white light-emitting devices.

Moreover, through the combination of the AIE characteristics and photoinduced singlet O_2_ production features of the metal clusters, imaging-guided potential photodynamic therapy (PDT) was realized [49,50]. First, the bioimaging performance of clusters **1**–**4** in live A549 cells was determined by confocal laser scanning microscopy. As shown in Fig. 5c and Supplementary Figs S29–S31, bright luminescence inside the cells was observed when the cells were incubated with the metal clusters for 15 min, indicating the good cell permeability of these clusters. The locations of clusters **1**–**4** in the cells were investigated by co-localization tests. The luminescence signals of the metal clusters overlapped well with those of LysoTracker deep red (a commercially available dye for lysosome staining), indicating that the clusters were located in the lysosomes. The co-localization coefficients of clusters **1**–**4** were as high as 94%, 97%, 91% and 93%, respectively. Because cluster **2** had the highest co-localization coefficient, its PDT application was explored in A549 cells by a standard cell counting kit-8 (CCK-8) assay. Dose-dependent toxicity experiments in the presence and absence of white light irradiation (5 mW/cm^2^) were carried out to determine the cell-killing efficiency of cluster **2**. As shown in Fig. 5d, cluster **2** exhibited high cytotoxicity under white light irradiation, and the viability of the A549 cells was <5% when the concentration of cluster **2** was 2 μmol/L. In contrast, nearly 98% viability was maintained in the cells incubated with 2 μmol/L cluster **2** in the dark. These results demonstrate the advantageous PDT property of cluster **2**.

## CONCLUSION

In conclusion, a series of copper clusters that exhibited intense colour-tuneable emissions in an oxygen-free environment or in an aggregated state were prepared. Based on the quenching of the phosphorescence of the metal clusters by oxygen, an ultrasensitive oxygen detection system was successfully constructed. Furthermore, unusual photoinduced emission enhancement in DMSO-containing environments was observed, leading to the first example of metal cluster-based photopatterning and photo-anticounterfeiting. More importantly, a new AIE mechanism, AIBO, was proposed, providing a new pathway to achieving the intense emission of metal clusters in the aggregated state. Intense emission in the aggregated state endowed the metal clusters that had cell imaging capability with a high co-localization coefficient and made the metal clusters suitable for the construction of light-emitting devices. This work not only reported a series of AIE metal clusters, illustrating their multiple applications, but, more importantly, also proposed a new AIE mechanism and provided a new perspective for understanding the influence of aggregation on the phosphorescence properties of molecules.

## Supplementary Material

nwab216_Supplemental_Files
