# Supplementary material for: Aggregation-induced barrier to oxygen—a new AIE mechanism for metal clusters with phosphorescence
Source: Natl Sci Rev. 2021 Nov 30;9(8):nwab216. doi: 10.1093/nsr/nwab216 (PMC9469893; doi:10.1093/nsr/nwab216)
Supplement: nwab216_Supplemental_Files [file nwab216_supplemental_files.zip › Revised_Supplementary_Materials.docx]

**Aggregation-induced barrier to oxygen (AIBO) - A new AIE mechanism for metal cluster with phosphorescence**

Yan Jin,^1^ Qiu-Chen Peng,^1^ Si Li,^1^ Hui Fang Su,^1^ Peng Luo,^1^ Ming Yang,^1^ Xin Zhang,^1^ Kai Li,^1,*^ Shuang-Quan Zang,^1,*^ Ben Zhong Tang^2,4*^ and Thomas C. W. Mak^1,3^

^1^ Henan Key Laboratory of Crystalline Molecular Functional Materials, Henan International Joint Laboratory of Tumor Theranostical Cluster Materials, Green Catalysis Center, and College of Chemistry, Zhengzhou University, Zhengzhou 450001, China

^2^ Shenzhen Institute of Aggregate Science and Technology, School of Science and Engineering, The Chinese University of Hong Kong, Shenzhen, Guangdong 518172, China

^3^ The Hong Kong Branch of Chinese National Engineering Research Center for Tissue Restoration and Reconstruction, The Hong Kong University of Science and Technology, Clear Water Bay, Kowloon, Hong Kong, China

^4^ Department of Chemistry, The Chinese University of Hong Kong, Shatin, New Territories, Hong Kong SAR, China

**Contents**

**1. Experimental........................................................................................................S2**

**1.1 Reagents..............................................................................................................S2**

**1.2 Apparatus............................................................................................................S2**

**1.3 Synthesis of the copper clusters..........................................................................S3**

**1.4 Preparation of PVDF film...................................................................................S4**

**1.5 Generating patterns on metal cluster contained PVDF film...........................S5**

**1.6 Preparation of 1 containing silica gel.................................................................S5**

**1.7 Luminescence measurement system for ultra-low concentration of oxygen...S6**

**1.8 Calculation Procedure........................................................................................S6**

**1.9 X-Ray Crystallography......................................................................................S7**

**1.10 Cell imaging and confocal colocalization.........................................................S7**

**1.11 ROS generation..................................................................................................S7**

**1.12 PDT study...........................................................................................................S8**

**1.13 References.........................................................................................................S8**

**2. Caption of video.................................................................................................S9**

**3. Selected spectra and data referred in the paper...............................................S10**

**1. Experimental**

**1.1 Reagents**

Unless otherwise noted, all materials used in this work are commercially available. [Cu(MeCN)_4_](PF_6_) and bis(diphenylphosphino)methane (dppm) were purchased from Energy Chemical, Shanghai, China. 4-Ethynylbenzonitrile was purchased from Leyan, Shanghai, China. 4-Ethynylpridedine, LysoTracker deep red, 2’,7’-dichlorodihydrofluorescein diacetate (DCFH-DA) and Cell Counting Kit-8 (CCK-8) were purchased from Sigma-Aldrich Chemical Co., Shanghai, China. 9-Ethynylphenanthrene was purchased from TCI, Shanghai, China. Polyvinylidene fluoride (PVDF) was purchased from J&K Chemical, Beijing, China. 9-(4-Ethynyl)carbazole was purchased from Zhengzhou Alfa Chemical Co., Ltd, Zhengzhou, China. Solvents of analytical grade were purchased from Sinopharm Chemical Reagent Beijing Co., Beijing, China.

**1.2 Apparatus**

Luminescence spectra, lifetimes and quantum yields of the solutions and solid samples were recorded on an Edinburgh FLS-980 fluorescence spectrometer. Luminescence spectra of film samples were recorded on a Hitachi F-4500 fluorescence spectrometer. Absorption spectra of the solutions and film samples were recorded on a Jasco V-750 UV-Vis spectrometer with a 1 cm quartz cell and a film sample holder, respectively. Ultraviolet diffuse reflection spectra of the solid samples were recorded on a Jasco V-750 UV-Vis spectrometer with an integrating sphere using a BaSO_4_ reference. NMR data were collected on a Bruker 400 Avance NMR spectrometer. DLS experiments were performed by means of a NanoPlus-3 dynamic light scattering particle size/zeta potential analyser at room temperature. Laser confocal scanning microscopy (LCSM) images were collected on a Zeiss laser scanning confocal microscope (LSM7 DUO). The photographs and videos are obtained by means of a NIKON D5500 camera.

**1.3 Synthesis of the copper clusters**

Trinuclear copper(I) clusters were prepared according to Yam et al [1]. The precursor [Cu_2_(μ-dppm)_2_(MeCN)_2_][PF_6_]_2_ and the corresponding alkyne ligand were used in the synthesis [2,3]. After stirring for 24 h at room temperature, the filtrate was diffused with diethyl ether, leading to the formation of crystals.

**Synthesis of 1**

A mixture of [Cu_2_(μ-dppm)_2_(MeCN)_2_][PF_6_]_2_ (0.033 g, 0.026 mmol) and 4-ethynylbenzonitrile (0.005 g, 0.039 mmol) in the presence of an excess of KOH in THF (4 mL) was stirred at room temperature for 24 h. After filtering, the filtrate was diffused with diethyl ether to obtain yellow crystals of **1**. Yield: 75%, based on [Cu_2_(μ-dppm)_2_(MeCN)_2_][PF_6_]_2_.

**Synthesis of 2**

The procedure was similar to that for **1** except 4-ethynylpyridine (0.004 g, 0.039 mmol) was used in place of 4-ethynylbenzonitrile. Yield: 73%, based on [Cu_2_(μ-dppm)_2_(MeCN)_2_][PF_6_]_2_.

**Synthesis of 3**

The procedure was similar to that for **1** except 9-(4-ethynyl)carbazole (0.011 g, 0.041 mmol) was used in place of 4-ethynylbenzonitrile. Yield: 63%, based on [Cu_2_(μ-dppm)_2_(MeCN)_2_][PF_6_]_2_.

**Synthesis of 4**

The procedure was similar to that for **1** except 9-ethynylphenanthrene (0.008 g, 0.039 mmol) was used in place of 4-ethynylbenzonitrile. Yield: 63%, based on [Cu_2_(μ-dppm)_2_(MeCN)_2_][PF_6_]_2_.

**Synthesis of 5**

The ligand *N*-(1-phenylethyl)-4-ethynylphthalimide was prepared according to a previously reported method [4]. 4-Ethynylphthalic anhydride (0.86 g, 5 mmol) and 1-phenylethylamine (0.73 g, 6 mmol) were stirred under reflux in acetic acid (15 mL) under a nitrogen atmosphere for 3 h at 120 °C. The crude product was chromatographed on silica gel with hexane/CH_2_Cl_2_ (*v*:*v* = 1:1) as the eluent. After purification, 1.15 g (84%) of *N*-(1-phenylethyl)-4-ethynylphthalimide was obtained. *N*-(1-Phenylethyl)-4-ethynylphthalimide (0.011 g, 0.04 mmol) was first dissolved in 3 mL MeOH and 3 mL THF, and Cu(MeCN)_4_PF_6_ (0.015 g, 0.04 mmol) was then added, causing the solution to change from clear to turbid orange-red under vigorous stirring. The resultant solution was treated with dppm until the solution was clear. After evaporation in the dark at room temperature, orange plate crystals were obtained, accompanied by by-product alkynyl ligand crystals. Yield: 60%, based on Cu(MeCN)_4_PF_6_.

**1.4 Preparation of** **metal cluster contained PVDF film**

PVDF films were prepared by thermally induced phase separation. First, 1 g PVDF and 0.2 g metal clusters were dissolved in a mixed solution containing 8 g *N,N*-dimethylformamide (DMF) and 0.8 g DMSO to prepare a casting solution with a metal cluster mass fraction of 2%. Then, the prepared casting solution was stirred at room temperature for 30 min to make it clear and viscous. After standing and degassing, the clear casting solution was evenly cast on a clean glass plate and dried under vacuum at 60 °C for 1 h. Finally, the formed transparent film was removed from the vacuum drying oven and peeled off carefully (Fig. S1). Thermogravimetric analysis (TGA) was used to investigate the thermostability of the metal clusters. As shown in Fig. S2, TGA curves suggested that all of **1**-**4** are stable 100 ^o^C, ensuring the integrity of the cluster in PVDF films.


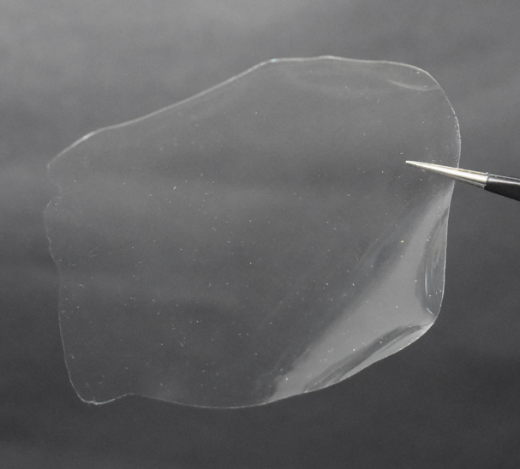


**Figure S1.** Photograph of the PVDF film under daylight.


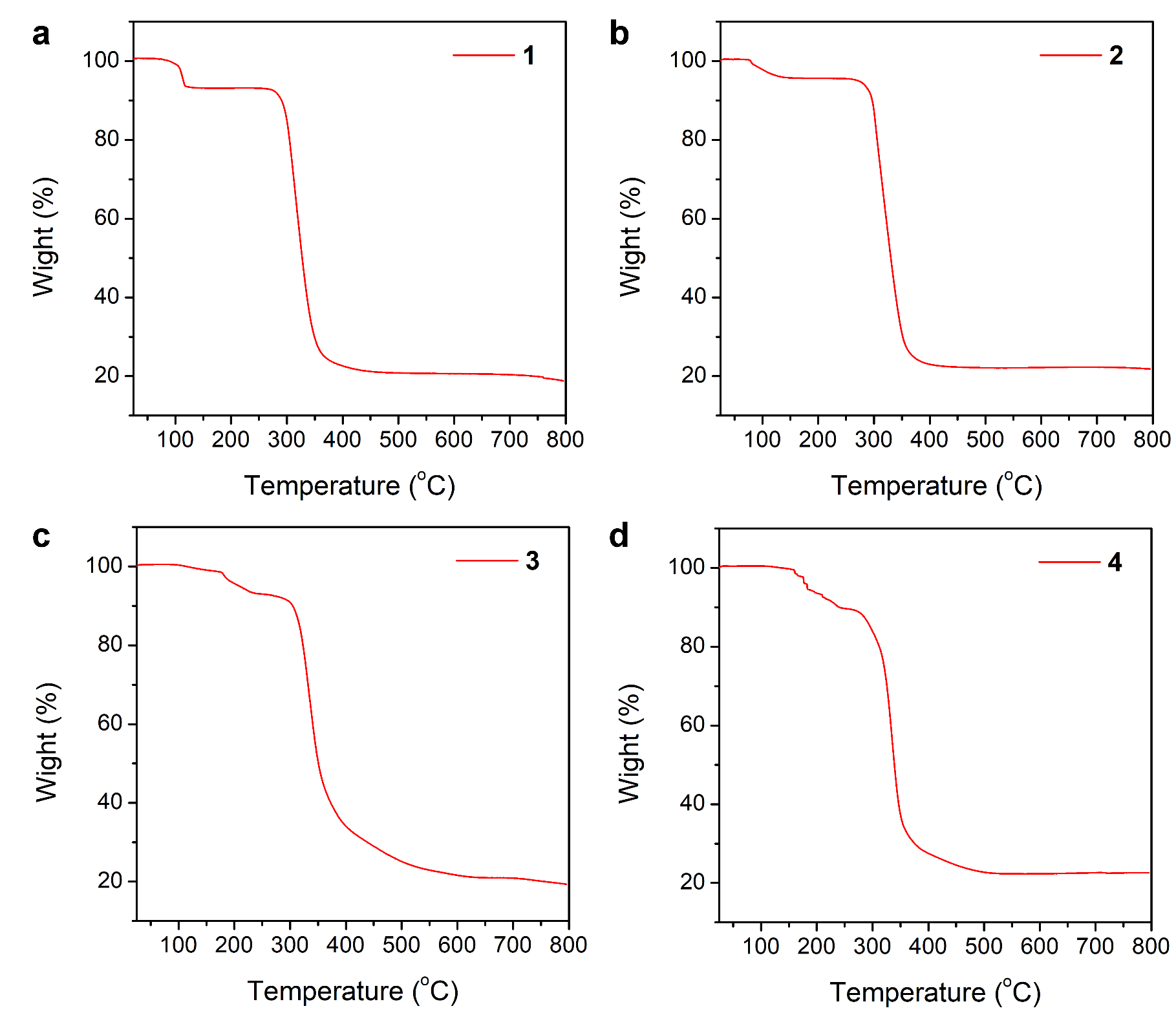


**Figure S2.** TGA curves of **1**-**4**.

**1.5 Generating patterns on metal cluster contained PVDF film**

As shown in Fig. S3, a pre-organized pattern was printed on transparencies, UV light was allowed to pass through the transparencies onto the metal cluster-containing PVDF film, and the patterns were subsequently recorded.


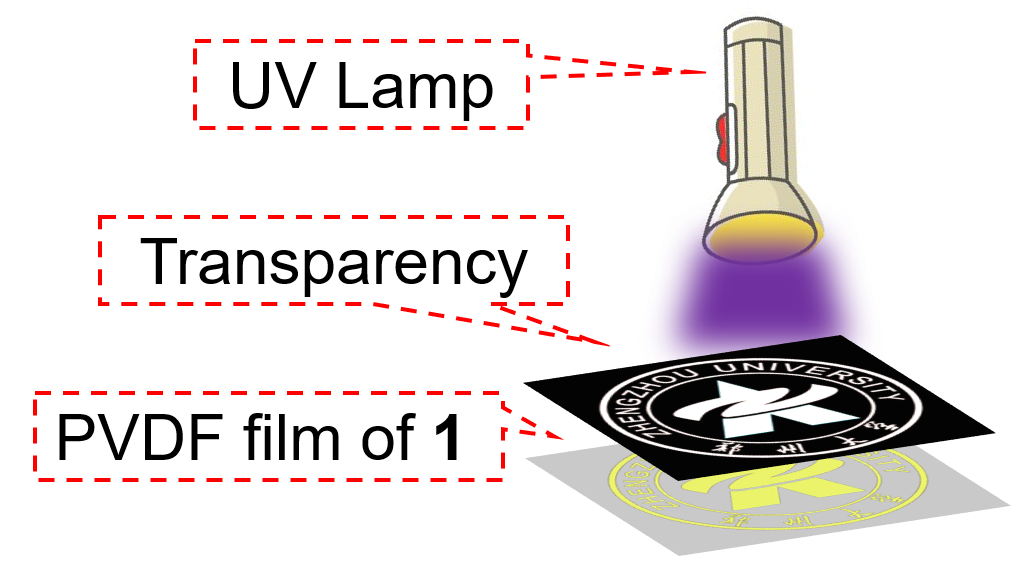


**Fig. S3.** A schematic diagram of the pattern generated on the metal cluster-containing PVDF film.

**1.6 Preparation of 1 containing silica gel**

First, 50 mg **1** was dissolved in 10 mL DCM, and then 10 g silica gel (300 mesh) was added. After mixing well, the DCM was removed under reduced pressure.

**1.7** **Luminescence measurement system for ultra-low concentration of oxygen**

*In situ* oxygen responses of photoluminescence were measured with a set-up that combined fluorescence signal collection and oxygen partial pressure control (Fig. S4) [5]. The fluorescence signal was collected through a Horiba Scientific FluoroMax-3 spectrofluorometer equipped with an FM4-3000 fibre-optic adaptor. The oxygen partial pressure was controlled by a 3H-2000Ps1 analysis instrument. The quartz container with samples was connected to the gas control instrument. The excitation light source was an optical fibre from a Xe lamp of the spectrophotometer. The emission signals were collected by the detector of the spectrophotometer through the other fibre bundle. Before injecting oxygen, the tube was evacuated for 1 h until the pressure was lower than 10^-3^ Pa. During the measurement, both bundles were covered by a light-shielding cloth.

**
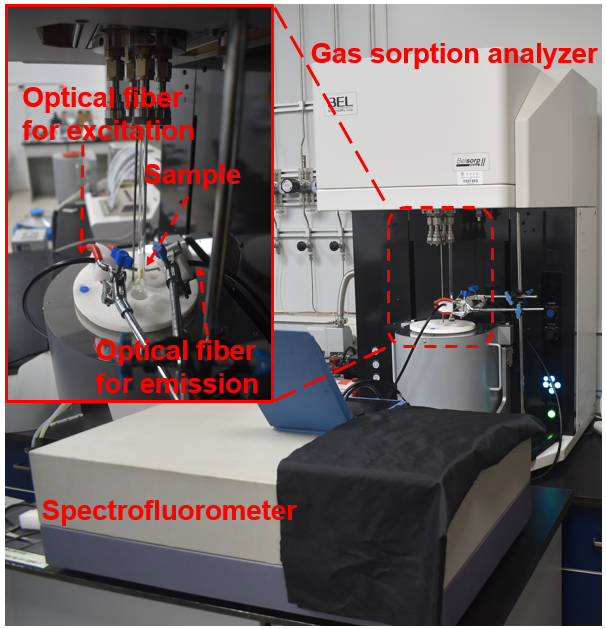
**

**Fig. S4.** Luminescence measurement system for ultra-low concentration of oxygen

**1.8 Calculation Procedure**

Density functional theory and time-dependent density functional theory calculations were performed with Gaussian 16 using the Perdew-Burke-Ernzerhof (PBE) functional [6,7]. All calculations were conducted using the 6-31G* basis set for H, C, N and P atoms [8,9] and Lanl2DZ effective core potentials for Cu atoms [10-12]. The single-crystal structures of **1-4** were chosen as the initial guess for ground-state optimization. Then, frequency calculations at the same level of theory were carried out to identify all of the stationary points as minima (zero imaginary frequency).

**1.9 X-Ray Crystallography**

Single-crystal X-ray diffraction measurements of the metal clusters were performed on a Rigaku XtaLAB Pro diffractometer with Cu-Kα radiation (λ = 1.5418 Å). Data collection and reduction were performed using the program CrysAlisPro [13]. All the structures were solved with direct methods (*SHELXS*) [14] and refined by full-matrix least squares on *F*^2^ using *OLEX2* [15], which utilized the *SHELXL*-2015 module [16]. All the atoms were refined anisotropically. Hydrogen atoms were placed in calculated positions refined using idealized geometries and assigned fixed isotropic displacement parameters. Structure refinement was performed with different strategies according to the electron density distribution. Detailed information about the crystal data and the refinement results for the clusters are summarized in Tables S1-S5.

**1.10 Cell imaging and confocal colocalization**

A549 cells were grown in a petri dish at 37.0 °C. The live cells were incubated with 5 μmol/L **1-4** or 1 μmol/L LysoTracker deep red for 15 min. Then, the dye-labelled A549 cells were imaged using a laser scanning confocal microscope. The wavelength of the laser was 488 nm, and the emission signals in the ranges of 550-650 nm and 650-740 nm were collected for cell imaging.

**1.11 ROS generation**

In the experiments, DCFH-DA was used as an indicator for ROS generation. DCFH-DA (5 μmol/L), 10 μmol/L **1-5** and a mixture of 10 μmol/L **1-5** and 5 μmol/L DCFH-DA were irradiated by 5 mW/cm^2^ white light in an aqueous solution or in DMSO. The emission at 534 nm was recorded at various irradiation times.

**1.12 PDT study of 2**

To evaluate the cytotoxicity of **2**, CCK-8 assays were used. A549 cells were seeded in a 96-well plate at a density of 6000-8000 cells per well. After cell growth for 12 h, the medium in each well was replaced with 100 mL fresh medium containing different concentrations of **2**. Then, some of the samples were treated with white light irradiation (5 mW/cm^2^, 10 min) and further incubated for 24 h. After that, 10 μL CCK-8 solution was added to each well. After incubation for 1 h, the absorption of each well (450 nm) was recorded *via* a Perkin-Elmer Victor3^TM^ plate reader.

**1.13** **References**

1. Yam V, Fung W and Wong M. Synthesis, photophysics, electrochemistry, and excited-state redox properties of trinuclear copper(I) acetylides with bis(diphenylphosphino)alkylamines and -arylamines as bridging ligands. *Organometallics* 1997; **16**: 1772-8.

2. Díez J, Gamasa M and Gimeno J et al. Binuclear copper(I) complexes containing bis(diphenylphosphino)methane bridging ligands: crystal structure of [Cu_2_(µ-dppm)_2_(MeCN)_4_][ClO_4_]_2_. *J Chem Soc Dalton Trans* 1987*;* **1***:* 1275-8.

3. Andrés-Tomé I, Winscom, C and Coppo, P. Copper(I) trinuclear phosphorescent complexes with tuneable optical and photophysical properties. *Eur J Inorg Chem* 2010*;* 3567-70.

4. Capitosti S, Hansen T and Brown M. Thalidomide analogues demonstrate dual inhibition of both angiogenesis and prostate cancer. *Bioorgan Med Chem* 2004*;* **12***:* 327-36*.*

5. Dong X, Si Y and Yang J et al. Ligand engineering to achieve enhanced ratiometric oxygen sensing in a silver cluster-based metal-organic framework. *Nat Commun* 2020; **11**: 3678.

6. Frisch M. Gaussian, Inc., Wallingford CT, 2009.

7. Perdew J, Burke K and Ernzerhof M. Generalized gradient approximation made simple. *Phys Rev Lett* 1996; **77**: 3865-8.

8. Hariharan P and Pople J. The influence of polarization functions on molecular orbital hydrogenation energies. *Theoret Chimica Acta* 1973; **28**: 213-22.

9. Francl M, Pietro W and Hehre W. Self‐consistent molecular orbital methods. XXIII. A polarization‐type basis set for second‐row elements. *J Chem Phys* 1982; **77**: 3654-65.

10. Hay P and Wadt W. Ab initio effective core potentials for molecular calculations. Potentials for the transition metal atoms Sc to Hg. *J Chem Phys* 1985; **82**: 270-83.

11. Hay P and Wadt W. Ab initio effective core potentials for molecular calculations. Potentials for main group elements Na to Bi. *J Chem Phys* 1985; **82**: 284-98.

12. Hay P and Wadt W. Ab initio effective core potentials for molecular calculations. Potentials for K to Au including the outermost core orbitals. *J Chem Phys* 1985; **82**: 299-310.

13. CrysAlisPro 2012, Agilent Technologies. Version 1.171.36.31.

14. Sheldrick G. A short history of SHELX. *Acta Cryst A* 2008; **64:** 112-22.

15. Dolomanov O, Bourhis L and Gildea R et al. OLEX2: a complete structure solution, refinement and analysis program. *J Appl Cryst* 2009; **42:** 339-41.

16. Sheldrick, G. Crystal structure refinement with SHELXL. *Acta Cryst C* 2015; **71:** 3-8.

**2. Caption of videos**

**Video 1.** Emission change of 50 μmol/L **1-4** in DMSO upon UV light irradiation.

**Video 2.** Pattern recording on PVDF film.

**Video 3. 1** in silica gel were exposed under alternating air/vacuum.

**3.** **Selected spectra and data referred in the paper**


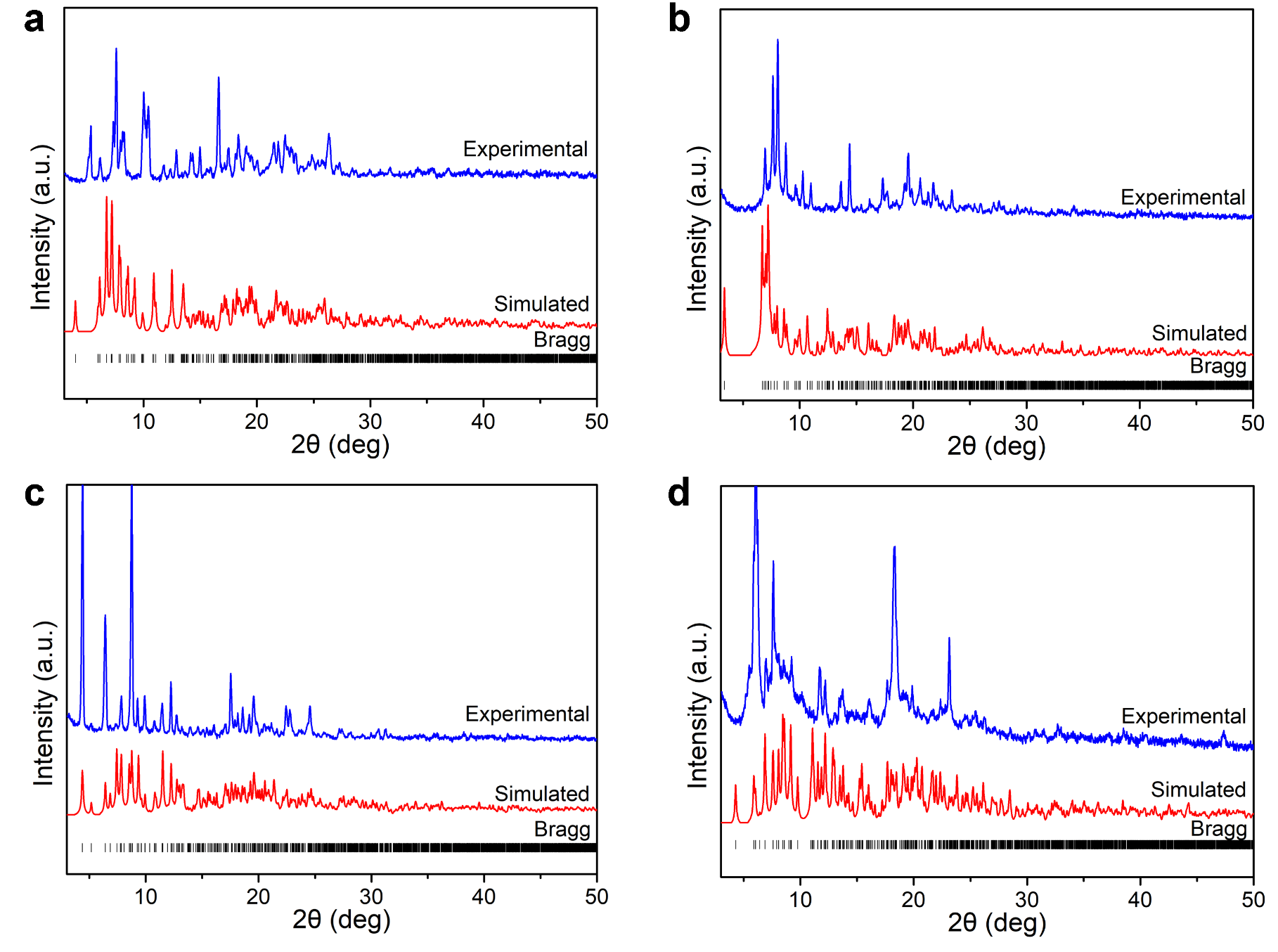


**Figure S5.** (a)-(d) PXRD patterns of **1**-**4**.


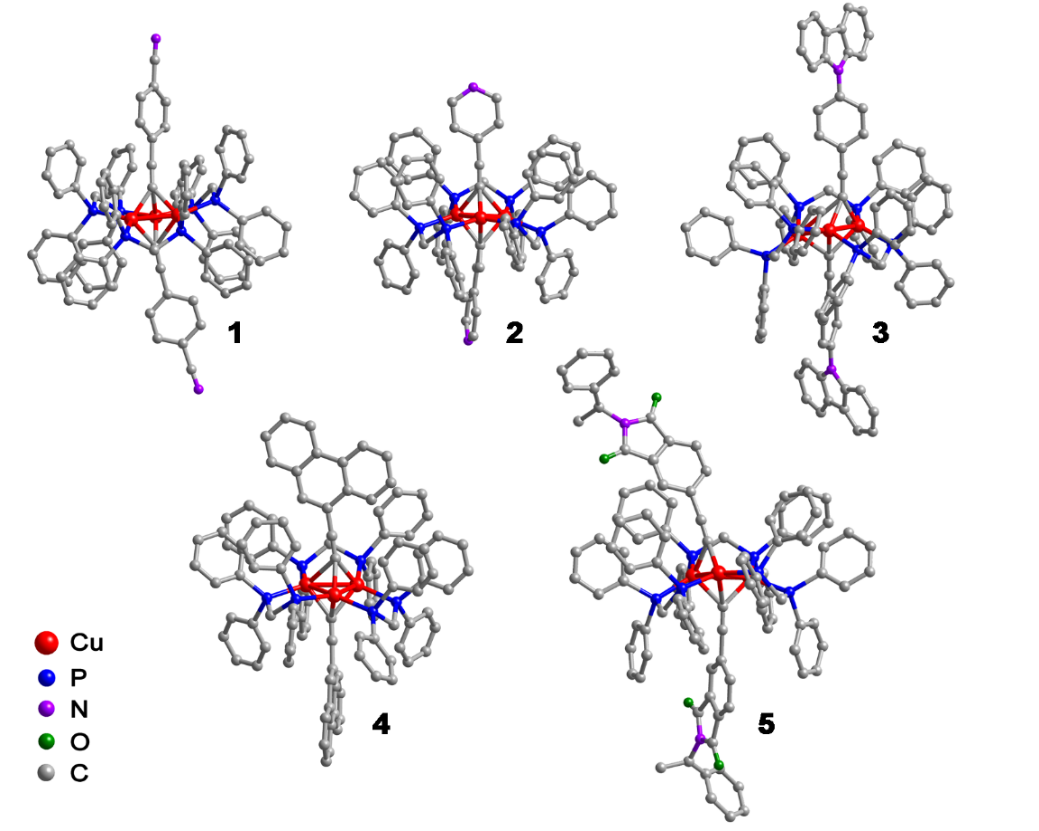


**Figure S6.** Crystal structures of **1**-**5**.


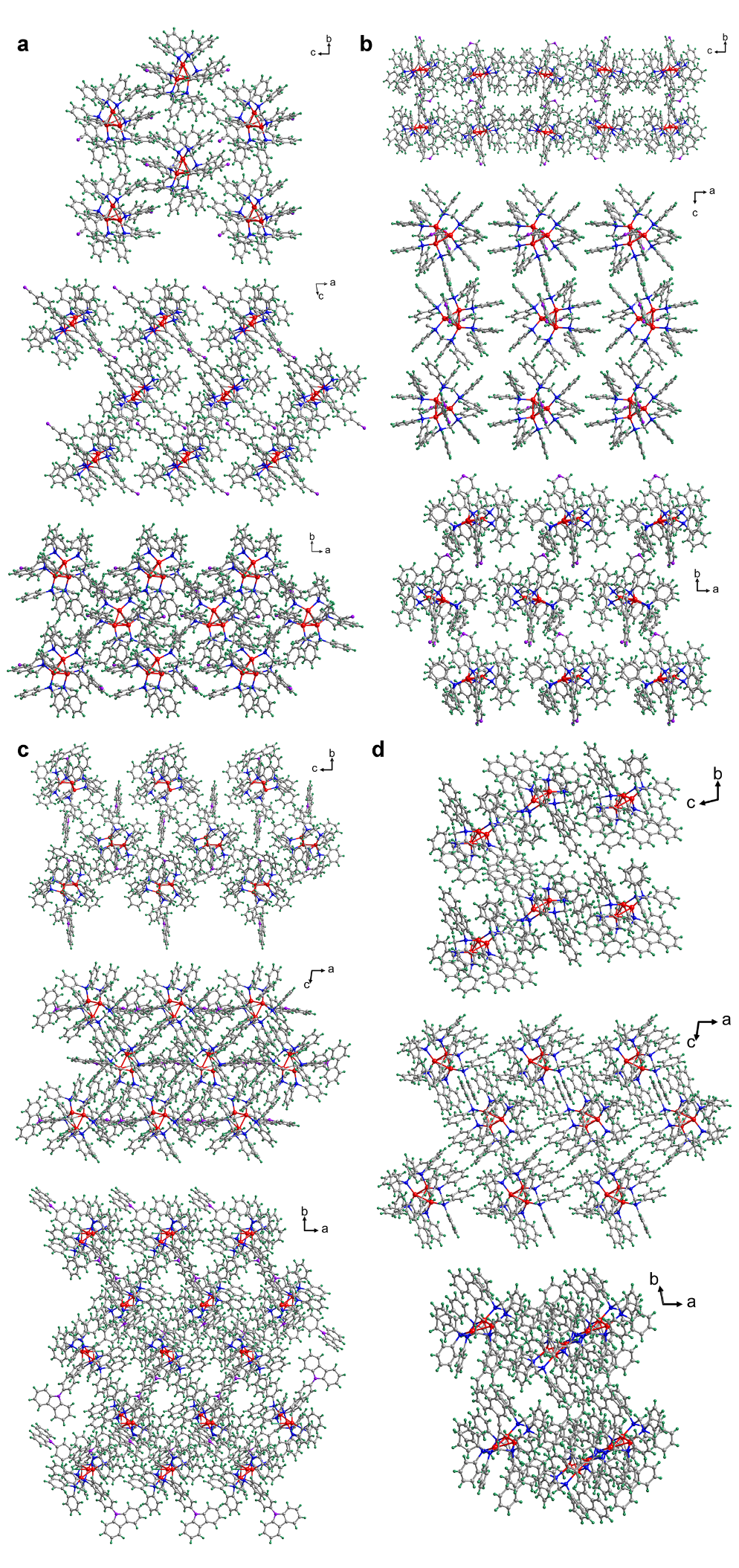


**Figure S7**. (a)-(d) The stacking patterns of **1**-**4** viewed from different directions.


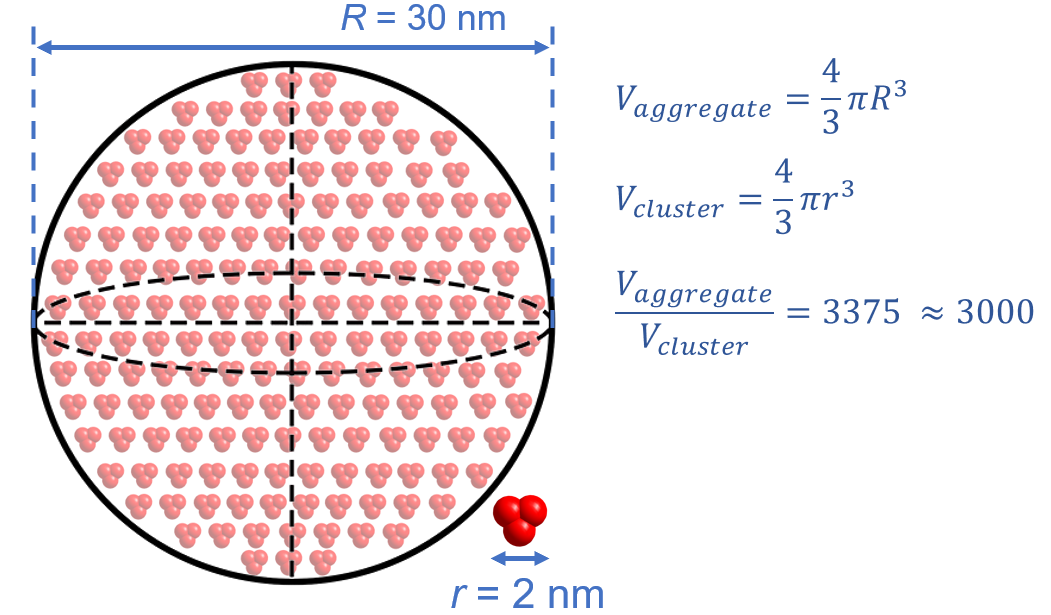


**Figure S8.** Schematic diagram of the smallest aggregate to express AIBO.


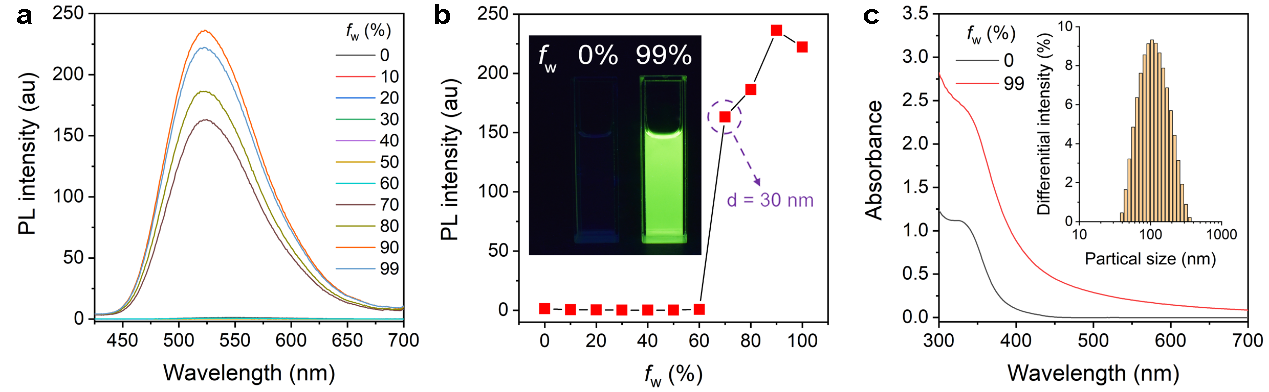


**Figure S9.** (a) Luminescence spectra of **2** in H_2_O/EtOH mixtures with different *f*_w_. (b) Luminescence intensity of **2** at 518 nm as a function of *f*_w_. Inset: Luminescence photo of **2** in H_2_O/EtOH mixtures with different *f*_w_. (c) Absorption spectra of **2** in H_2_O/EtOH mixtures with different *f*_w_. Inset: DLS results of **2** in 99% H_2_O/EtOH mixture. Condition: [**2**] = 50 μmol/L.


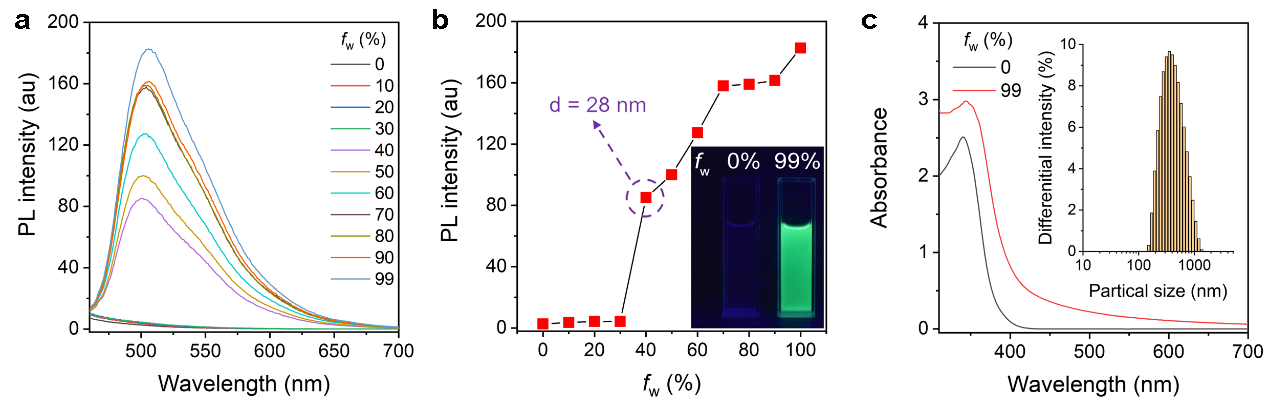


**Figure S10.** (a) Luminescence spectra of **3** in H_2_O/EtOH mixtures with different *f*_w_. (b) Luminescence intensity of **3** at 505 nm as a function of *f*_w_. Inset: Luminescence photo of **3** in H_2_O/EtOH mixtures with different *f*_w_. (c) Absorption spectra of **3** in H_2_O/EtOH mixtures with different *f*_w_. Inset: DLS results of **3** in 99% H_2_O/EtOH mixture. Condition: [**3**] = 50 μmol/L.


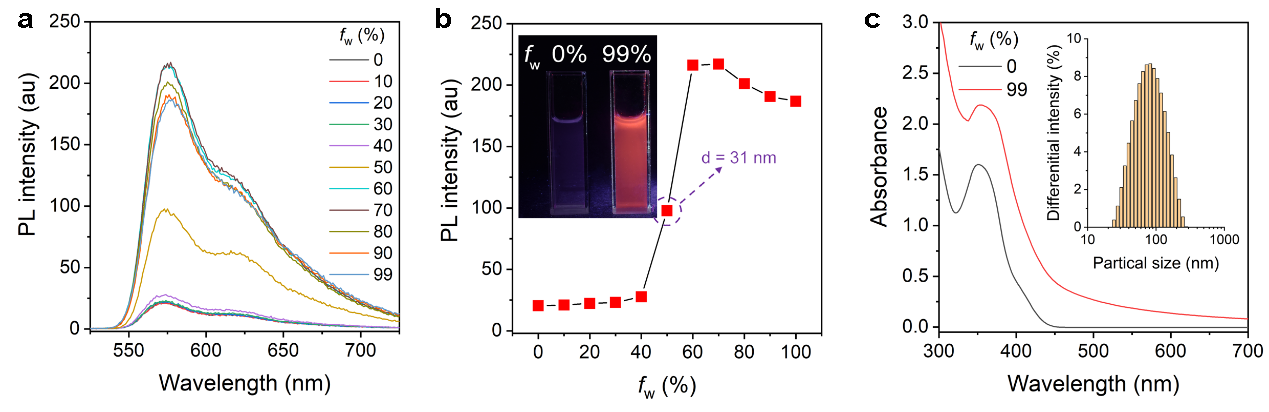


**Figure S11.** (a) Luminescence spectra of **4** in H_2_O/EtOH mixtures with different *f*_w_. (b) Luminescence intensity of **4** at 578 nm as a function of *f*_w_. Inset: Luminescence photo of **4** in H_2_O/EtOH mixtures with different *f*_w_. (c) Absorption spectra of **4** in H_2_O/EtOH mixtures with different *f*_w_. Inset: DLS results of **4** in 99% H_2_O/EtOH mixture. Condition: [**4**] = 50 μmol/L.


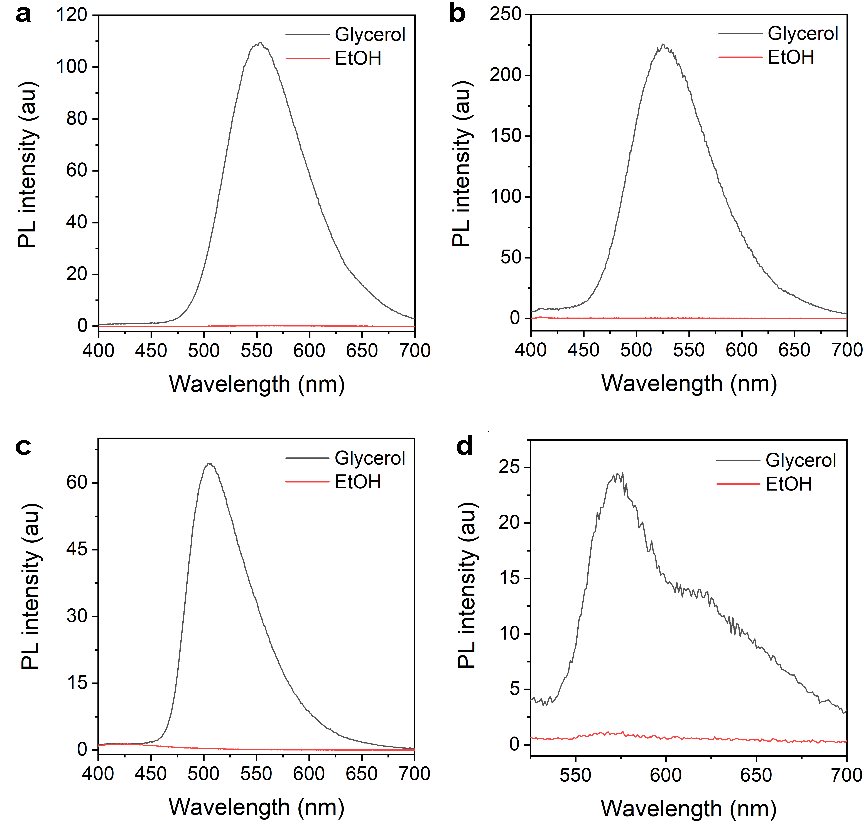


**Figure S12.** (a)-(d) Luminescence spectra of **1**-**4** in glycerol/EtOH mixtures with different *f*_g_.


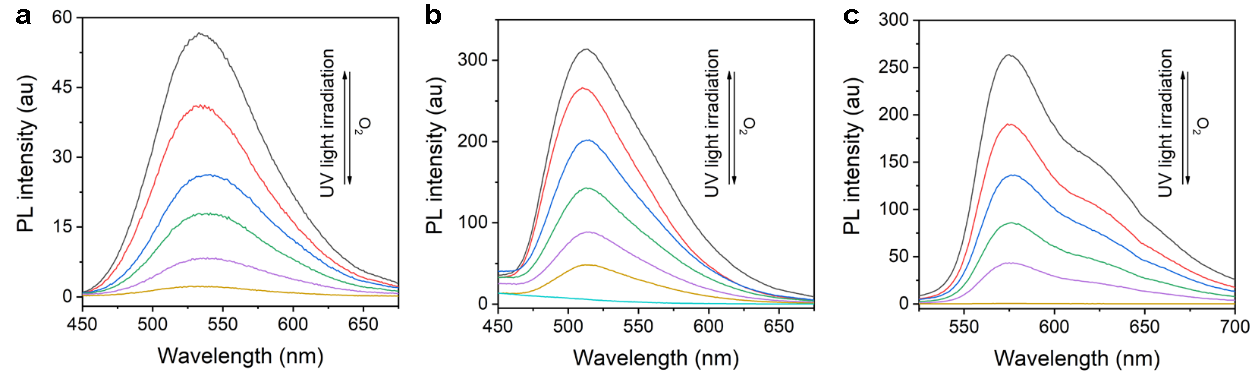


**Figure S13.** (a)-(c) Luminescence spectra of **2-4** in DMSO upon UV light irradiation. Condition: [**2**] = [**3**] = [**4**] = 50 μmol/L.


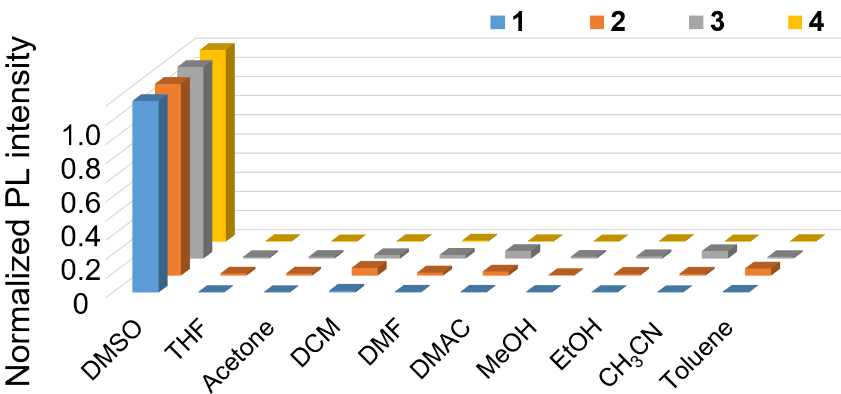


**Figure S14.** Normalized luminescence intensity of **1**-**4** after UV light irradiation (20 mW cm^-2^) for 5 min in different solvents. Condition: [**1-4**] = 50 μmol/L.


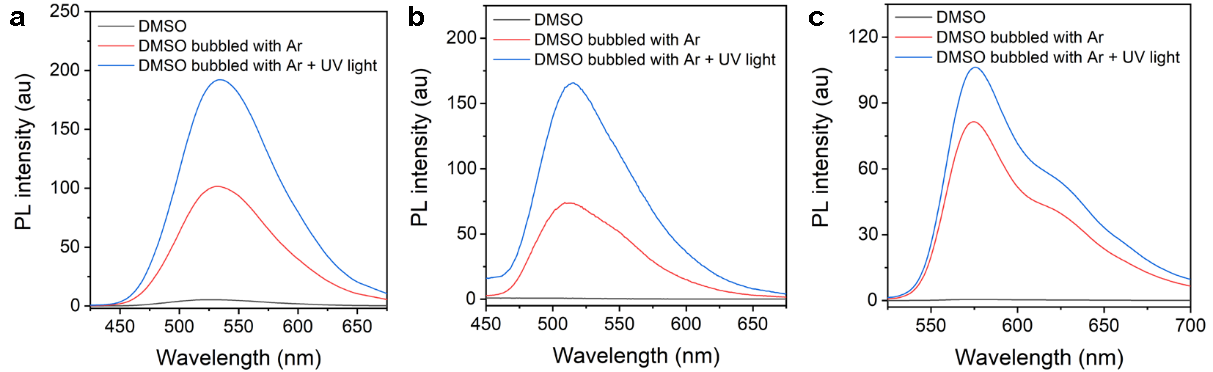


**Figure S15.** (a)-(c) Luminescence spectra of **2-4** in DMSO before and after bubbling with Ar, and irradiated with UV light. Condition: [**2**] = [**3**] = [**4**] = 50 μmol/L.


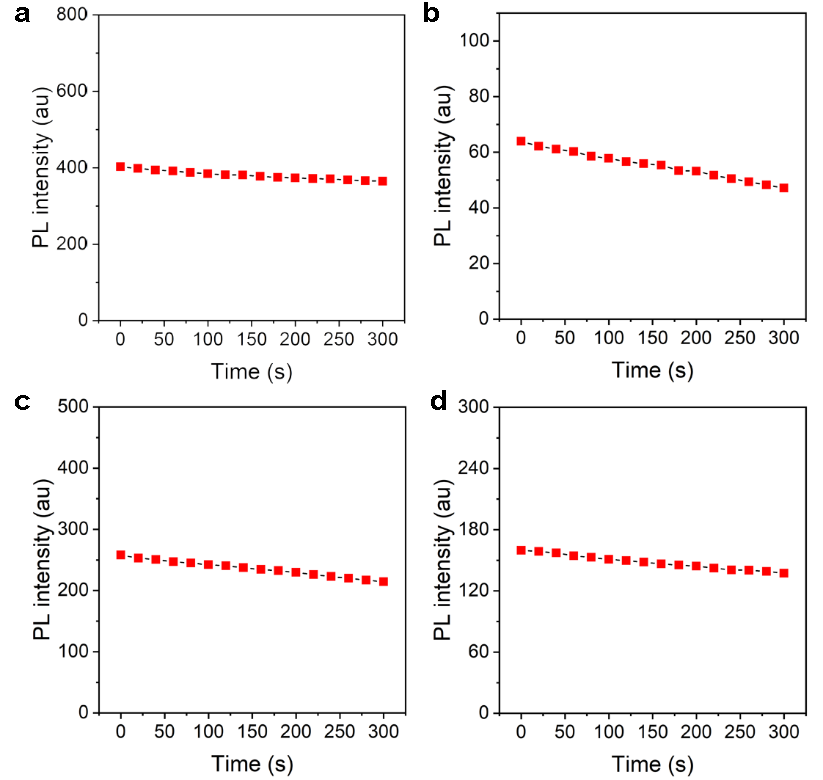


**Figure S16.** Time-dependent luminescence intensity of (a) **1** and 568 nm, (b) **2** at 529 nm, (c) **3** at 514 nm and (d) **4** at 572 nm in oxygen-free DMSO. Condition: [**1**] = [**2**] = [**3**] = [**4**] = 50 μmol/L.


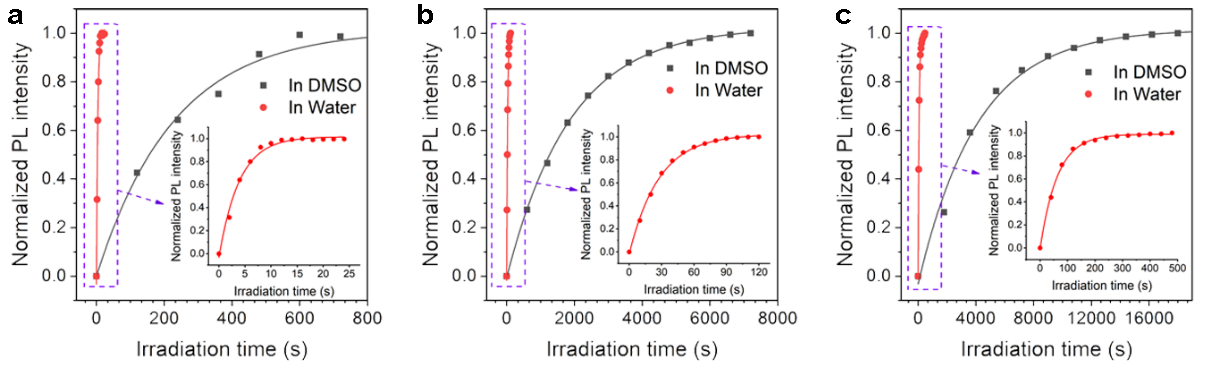


**Figure S17.** (a)-(c) Fluorescence intensity changes at 525 nm of 5 μmol/L DCFH-DA and 10 μmol/L **2-4** in aqueous solution and DMSO solution upon white light irradiation for different times. Condition: [**2**] = [**3**] = [**4**] = 50 μmol/L.


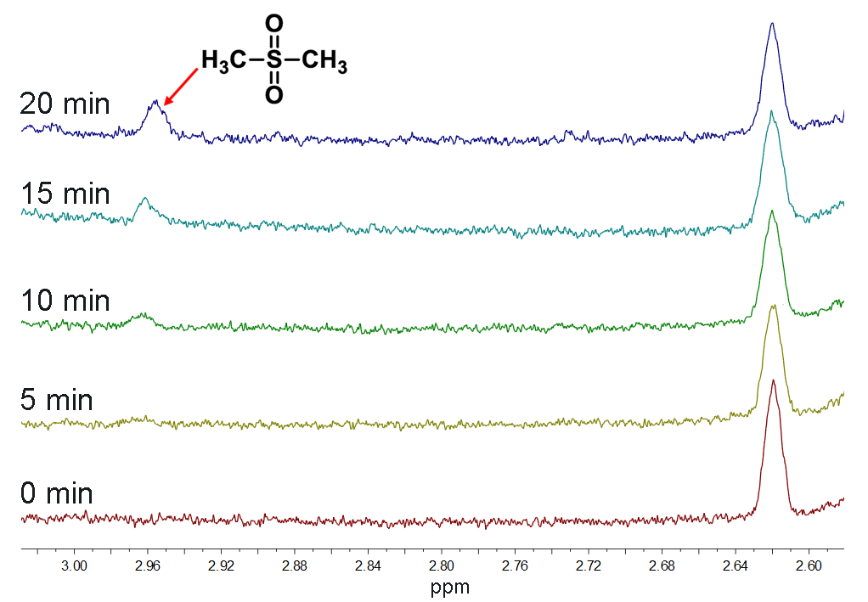


**Figure S18.** ^1^H-NMR spectra of **1** in DMSO-*d*_6_ upon UV light irradiation for different times.


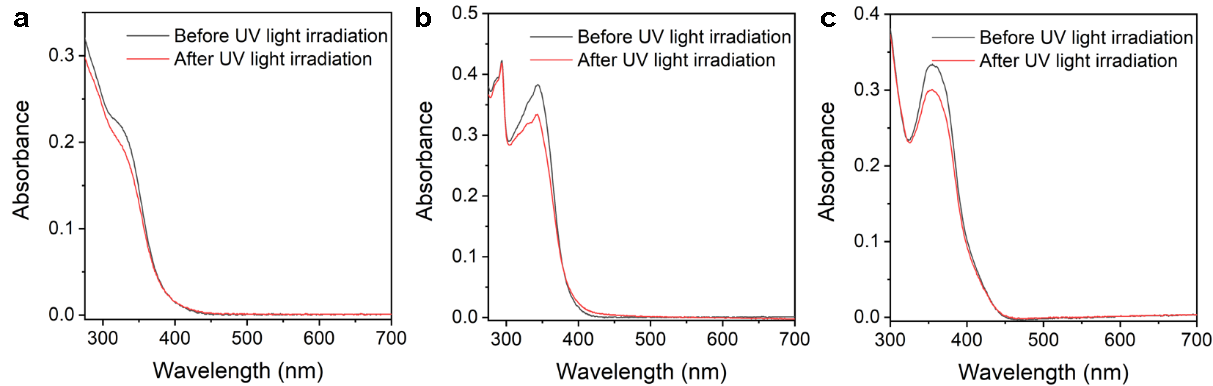


**Figure S19.** (a)-(c) Absorption spectra of **2-4** in DMSO before and after UV light irradiation. Condition: [**2**] = [**3**] = [**4**] = 50 μmol/L.


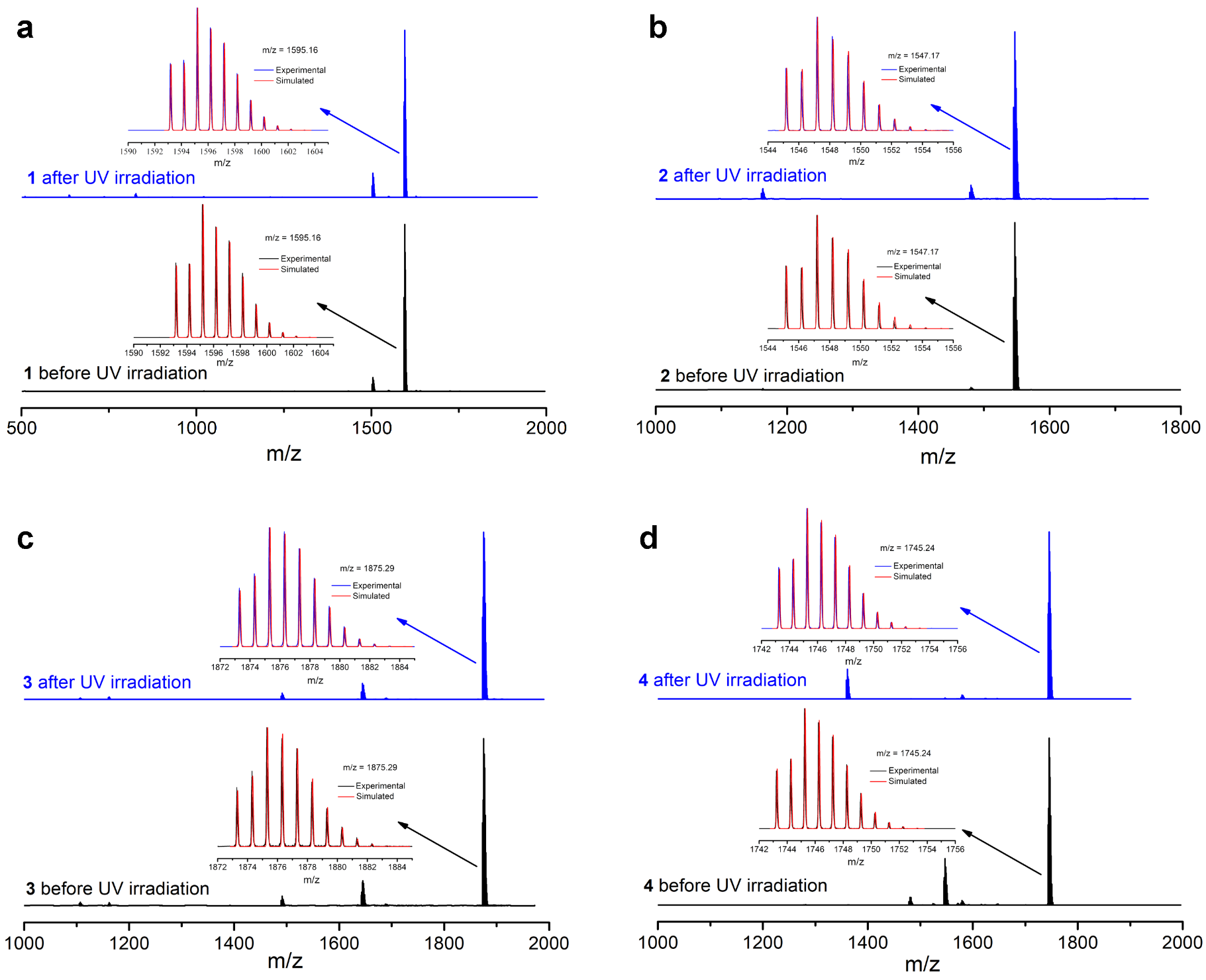


**Figure S20.** (a)-(d) HRMS of **1**-**4** in DMSO before and after UV light irradiation.


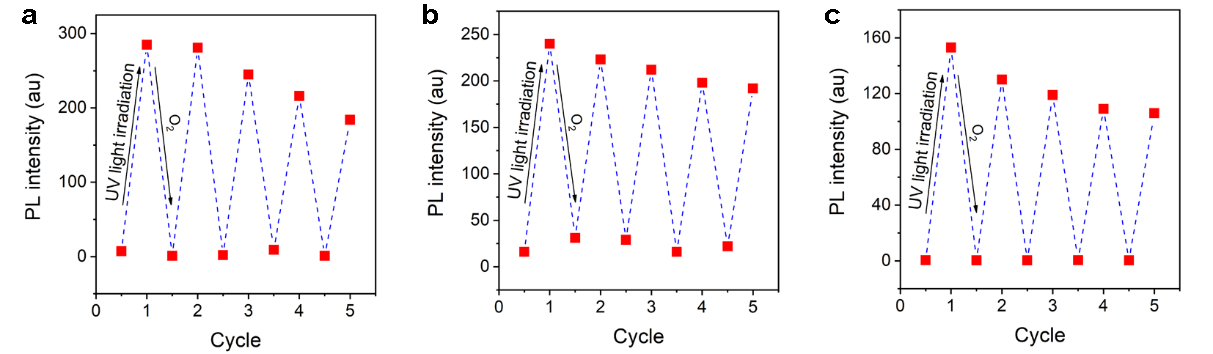


**Figure S21.** (a)-(c) Fatigue resistance of **2-4** upon UV light irradiation and standing in air alternately. Condition: [**2**] = [**3**] = [**4**] = 50 μmol/L.


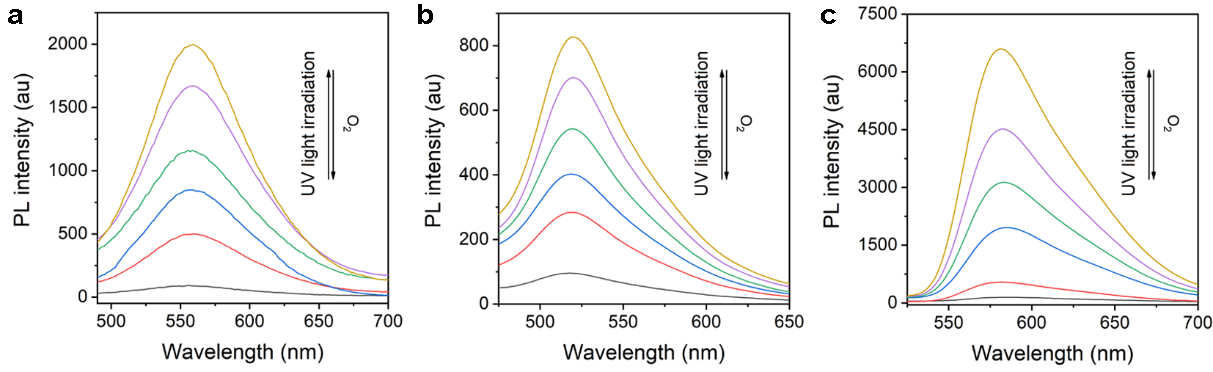


**Figure S22.** (a)-(c) Luminescence spectra of **2-4** in PVDF film before and after UV light irradiation.


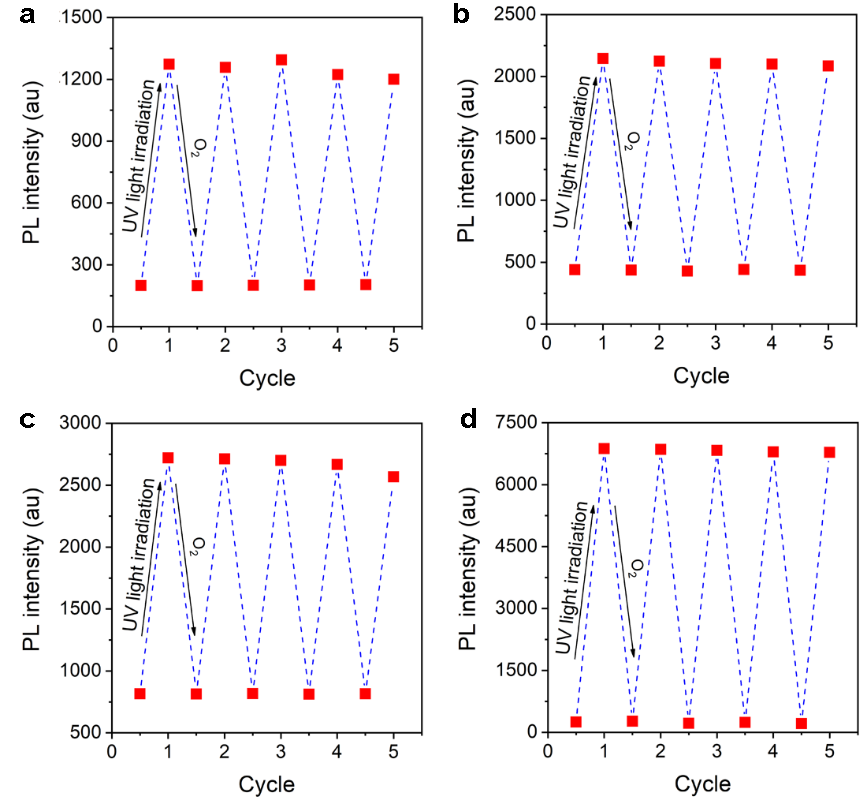


**Figure S23.** (a)-(d) Fatigue resistance of **1-4** in PVDF film upon UV light irradiation and standing in air alternately.


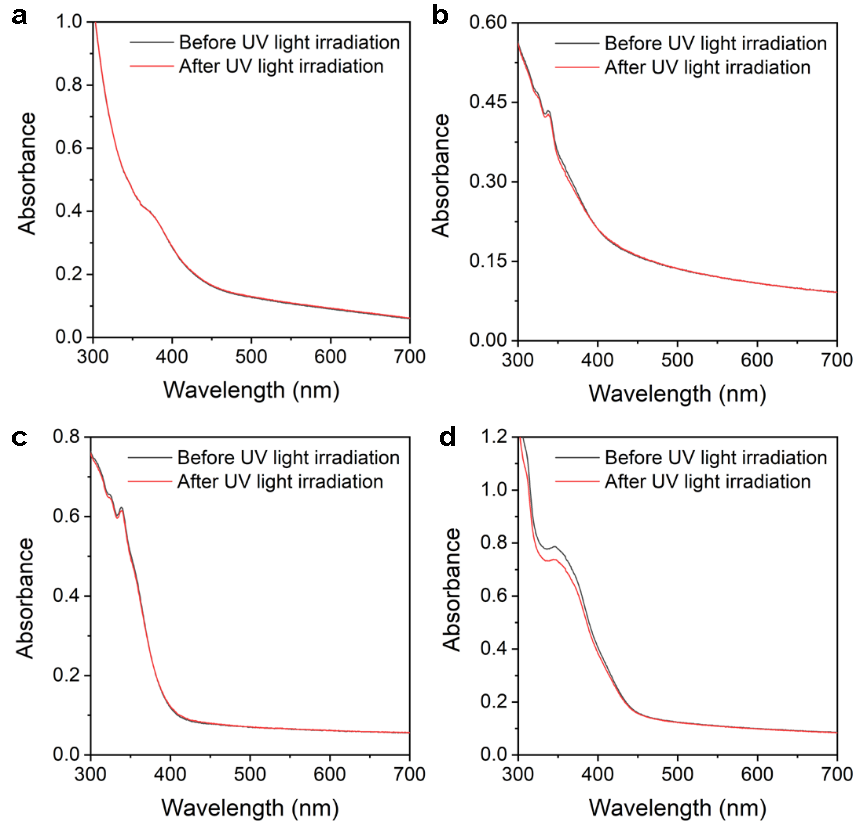


**Figure S24.** (a)-(d) Absorption spectra of **1-4** in PVDF film before and after UV light irradiation.


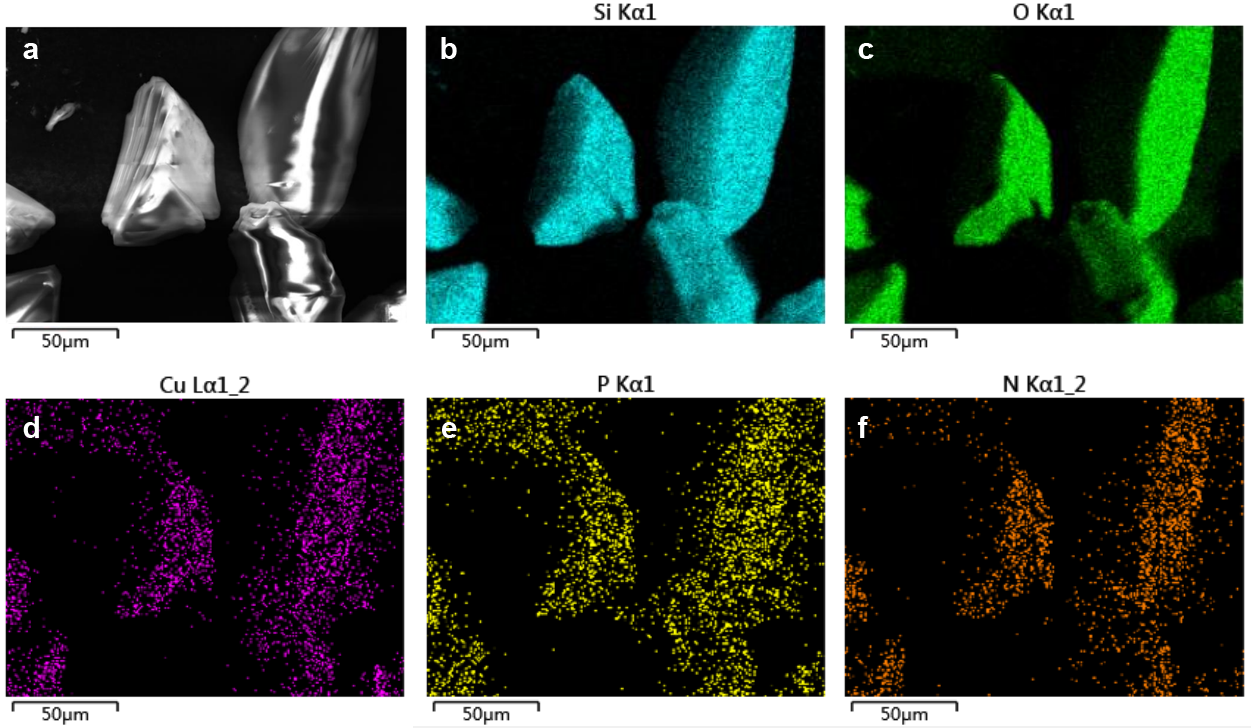


**Figure S25.** (a) SEM image and (b)-(f) elemental mapping of **1** on silica gel.


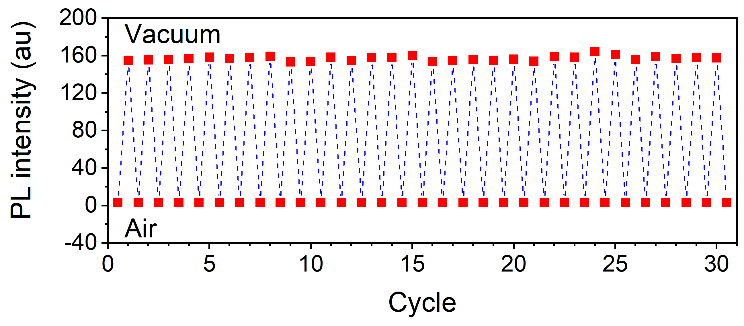


**Figure S26.** Reversible luminescence cycles of **1** in silica gel under alternating exposure to air/vacuum. Mass fraction of **1** in the sample was 0.5%.


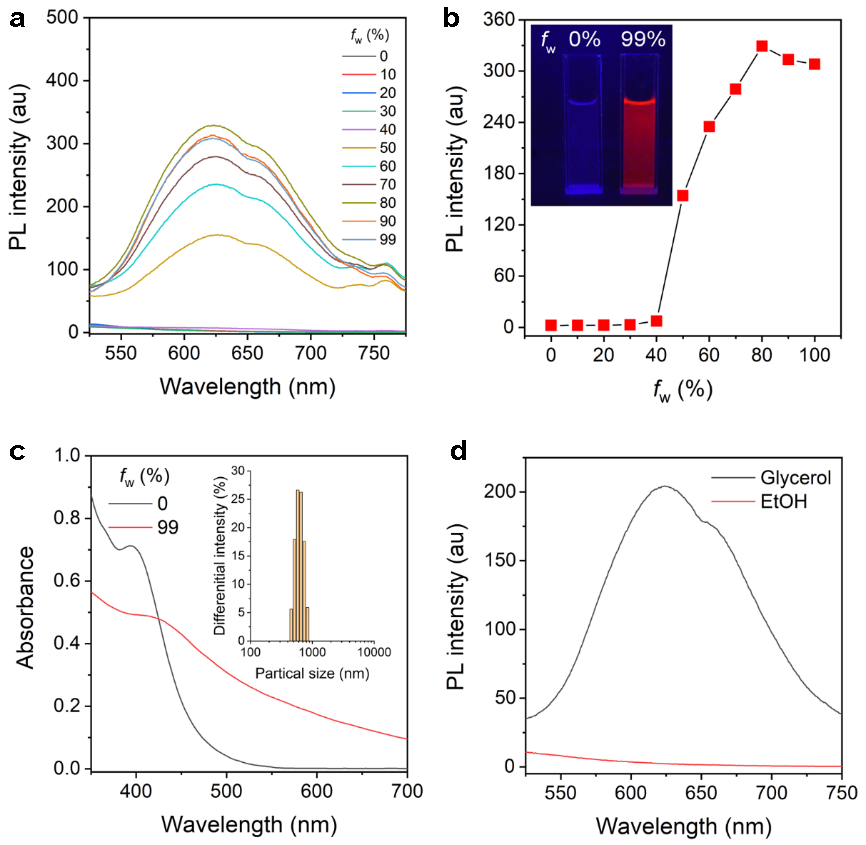


**Figure S27.** (a) Luminescence spectra of **5** in H_2_O/EtOH mixtures with different *f*_w_. (b) Luminescence intensity of **5** at 559 nm as a function of *f*_w_. Inset: Luminescence photo of **5** in H_2_O/EtOH mixtures with different *f*_w_. (c) Absorption spectra of **5** in H_2_O/EtOH mixtures with different *f*_w_. Inset: DLS results of **5** in 99% H_2_O/EtOH mixture. (d) Luminescence spectra of **5** in glycerol/EtOH mixtures with different *f*_g_. Condition: [**5**] = 50 μmol/L.


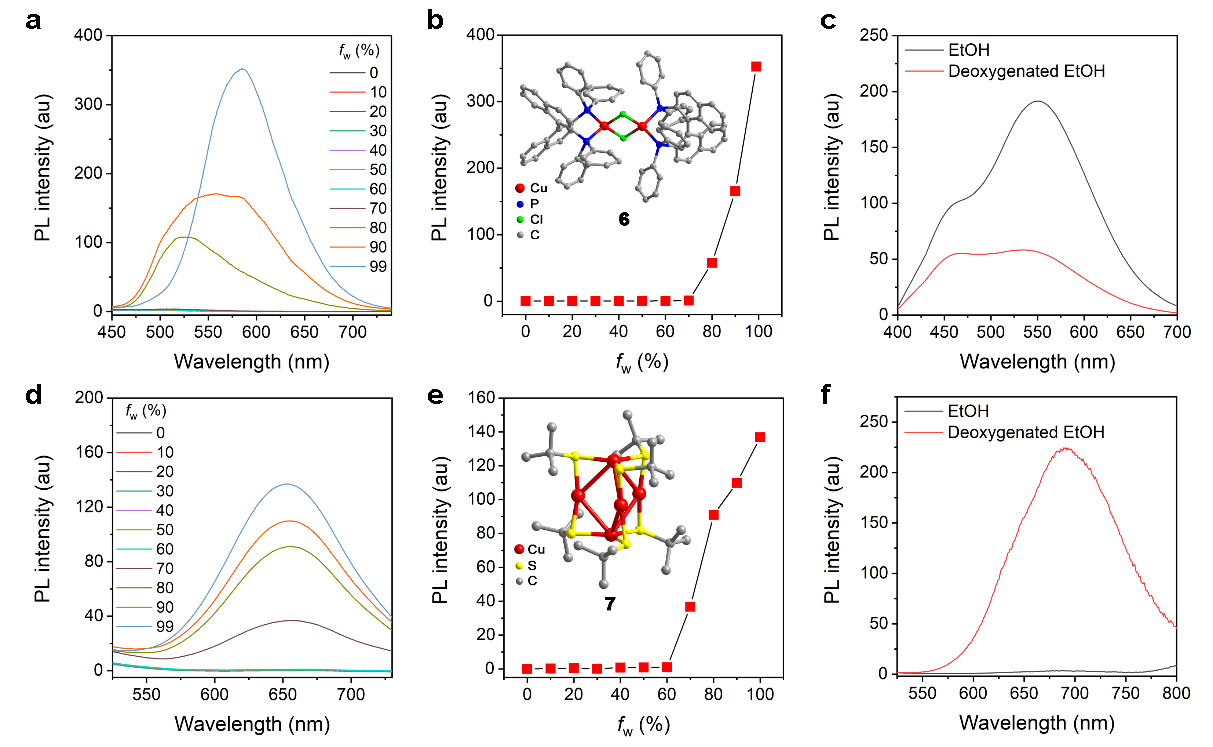


**Figure S28.** Luminescence spectra of (a) **6** and (d) **7** in solution before and after bubbling with N_2_. Condition: [**6**] = [**7**] =50 μmol/L. Luminescence spectra of (b) **6** and (e) **7** in H_2_O/EtOH mixtures with different *f*_w_. Luminescence intensity of (c) **6** at 585 nm and (f) **7** at 654 nm as a function of *f*_w_. Inset of (c) and (f) The crystal structures of **6** and **7**. Condition: [**6**] = [**7**] =50 μmol/L.


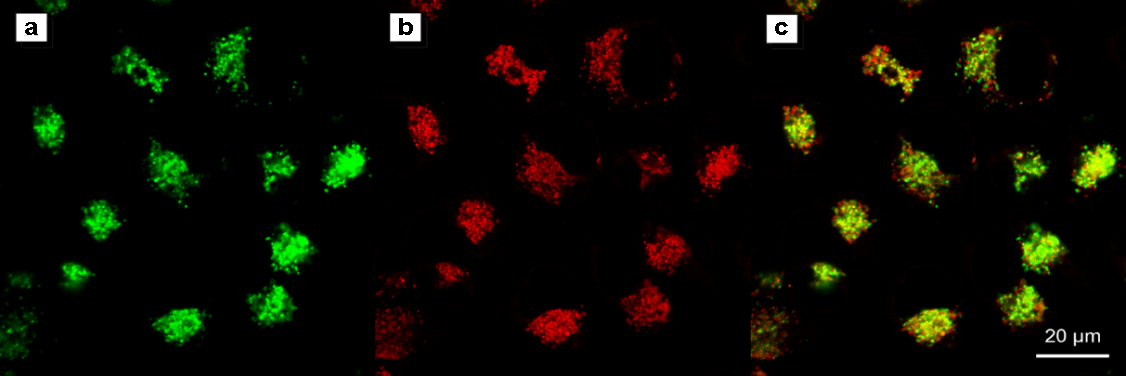


**Figure S29.** Colocalization imaging of A549 cells stained with (a) 5 µmol/L **1** (green, 500-650 nm) and (b) 0.1 µmol/L Lysotracker deep red (red, 650-740 nm). (c) Merged image of panels (a) and (b).


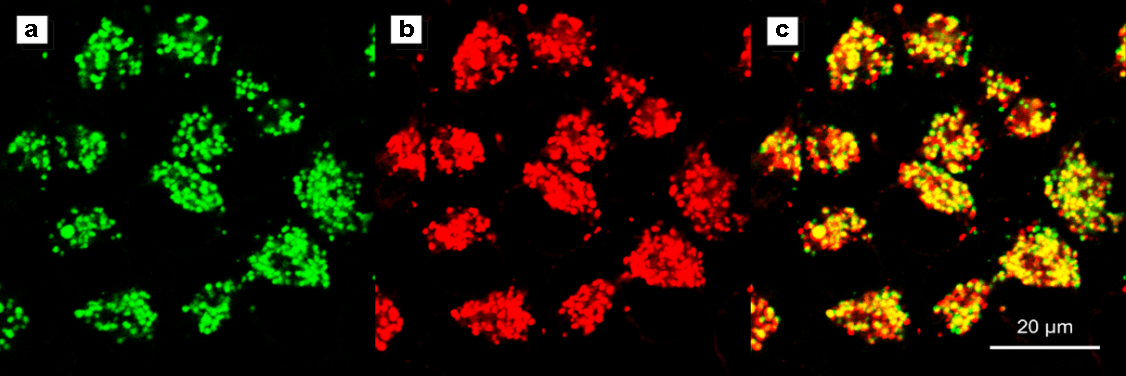


**Figure S30.** Colocalization imaging of A549 cells stained with (a) 5 µmol/L **3** (green, 500-650 nm) and (b) 0.1 µmol/L Lysotracker deep red (red, 650-740 nm). (c) Merged image of panels (a) and (b).


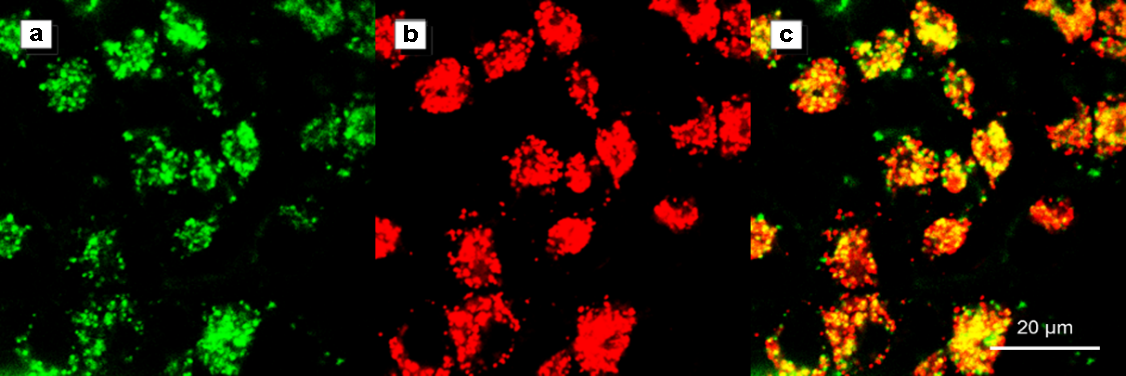


**Figure S31.** Colocalization imaging of A549 cells stained with (a) 5 µmol/L **4** (green, 530-650 nm) and (b) 0.1 µmol/L Lysotracker deep red (red, 650-740 nm). (c) Merged image of panels (a) and (b).

**Table S1.** Time for light up the phosphorescence of **1**-**4** by UV light irradiation in DMSO and Δ*E*_s1-t1_ of **1**-**4**.

|  | **1** | **2** | **3** | **4** |
| --- | --- | --- | --- | --- |
| Time for lighting up (s) | 5 | 5 | 11 | 2 |
| *E*_s1_ (eV) | 2.73 | 2.92 | 2.86 | 2.86 |
| *E*_t1_ (eV) | 2.46 | 2.70 | 2.48 | 2.73 |
| Δ*E*_s1-t1_ (eV) | 0.27 | 0.22 | 0.38 | 0.13 |

**Table S2.** QY of 50 μmol/L of **1**-**4** in deoxygenated DMSO and in glycerol.

|  | **1** | **2** | **3** | **4** | **5** |
| --- | --- | --- | --- | --- | --- |
| Deoxygenated DMSO | 33% | 29% | 48% | 28% | ~0% |
| Glycerol | 3.9% | 6.2% | 4.3% | 1.2% | 3% |

**Table S3.** Crystal data and structure refinements of **1**.

|  | **1** |
| --- | --- |
| Empirical formula | C_101_H_93_Cu_3_F_6_N_2_O_2_P_7_ |
| Formula weight | 1888.18 |
| Temperature/K | 200.00(10) |
| Crystal system | triclinic |
| Space group | *P*1 |
| a/Å | 14.6187(3) |
| b/Å | 14.8046(2) |
| c/Å | 22.5114(3) |
| *α*/° | 89.3530(10) |
| *β*/° | 80.4580(10) |
| *γ*/° | 89.0400(10) |
| Volume/Å3 | 4803.75(13) |
| Z | 2 |
| ρcalcg/cm^3^ | 1.305 |
| *μ*/mm^‒1^ | 0.834 |
| F(000) | 1950.0 |
| Crystal size/mm^3^ | 0.08 × 0.06 × 0.06 |
| Radiation | MoKα (λ = 0.71073) |
| 2θ range for data collection/° | 5.506 to 58.292 |
| Index ranges | -19 ≤ h ≤ 19, -19 ≤ k ≤ 20, -29 ≤ l ≤ 30 |
| Reflections collected | 138229 |
| Independent reflections | 43072 [*R*_int_ = 0.0444, *R*_sigma_ = 0.0504] |
| Data/restraints/parameters | 43072/160/2184 |
| Goodness-of-fit on F^2^ | 1.045 |
| Final R indexes [I>=2σ (I)] | *R_1_* = 0.0492, *wR_2_* = 0.1302 |
| Final R indexes [all data] | *R_1_* = 0.0601, *wR_2_* = 0.1358 |
| CCDC | 2079693 |

*R_1_ = ∑׀׀F_o_׀−׀F_c_׀׀/∑׀F_o_׀. wR_2_ = [∑w(F_o_^2^ −F_c_^2^)^2^/∑w(F_o_^2^)^2^]^1/2^*

**Table S4.** Crystal data and structure refinements of **2**.

|  | **2** |
| --- | --- |
| Empirical formula | C_89_H_74_Cu_3_F_6_N_2_P_7_ |
| Formula weight | 1692.91 |
| Temperature/K | 200.00(10) |
| Crystal system | orthorhombic |
| Space group | *Pbcn* |
| a/Å | 14.79000(10) |
| b/Å | 24.3336(2) |
| c/Å | 52.8541(6) |
| *α*/° | 90 |
| *β*/° | 90 |
| *γ*/° | 90 |
| Volume/Å3 | 19021.9(3) |
| Z | 8 |
| ρcalcg/cm^3^ | 1.181 |
| *μ*/mm^‒1^ | 2.311 |
| F(000) | 6944.0 |
| Crystal size/mm^3^ | 0.07 × 0.06 ×0.06 |
| Radiation | CuKα (λ = 1.54184) |
| 2θ range for data collection/° | 6.848 to 146.596 |
| Index ranges | -18 ≤ h ≤ 17, -26 ≤ k ≤ 30, -64 ≤ l ≤ 63 |
| Reflections collected | 46947 |
| Independent reflections | 18432 [*R*_int_ = 0.0346, *R*_sigma_ = 0.0412] |
| Data/restraints/parameters | 18432/657/1056 |
| Goodness-of-fit on F^2^ | 1.069 |
| Final R indexes [I>=2σ (I)] | *R_1_*= 0.1268, *wR_2_* = 0.3169 |
| Final R indexes [all data] | *R_1_*= 0.1334, *wR_2_* = 0.3209 |
| CCDC | 2079697 |

*R_1_ = ∑׀׀F_o_׀−׀F_c_׀׀/∑׀F_o_׀. wR_2_ = [∑w(F_o_^2^ −F_c_^2^)^2^/∑w(F_o_^2^)^2^]^1/2^*

**Table S5.** Crystal data and structure refinements of **3**.

|  | **3** |
| --- | --- |
| Empirical formula | C_123_H_106_Cu_3_F_6_N_2_O_2_P_7_ |
| Formula weight | 2165.50 |
| Temperature/K | 200.00(10) |
| Crystal system | monoclinic |
| Space group | *P*2_1_/*n* |
| a/Å | 13.76900(10) |
| b/Å | 40.3432(3) |
| c/Å | 19.1026(2) |
| *α*/° | 90 |
| *β*/° | 98.3310(10) |
| *γ*/° | 90 |
| Volume/Å3 | 10499.24(16) |
| Z | 4 |
| ρcalcg/cm^3^ | 1.370 |
| *μ*/mm^‒1^ | 2.234 |
| F(000) | 4480.0 |
| Crystal size/mm^3^ | 0.07 × 0.06 × 0.06 |
| Radiation | CuKα (λ = 1.54184) |
| 2θ range for data collection/° | 5.164 to 146.574 |
| Index ranges | -16 ≤ h ≤ 16, -35 ≤ k ≤ 49, -18 ≤ l ≤ 23 |
| Reflections collected | 45274 |
| Independent reflections | 20225 [*R*_int_ = 0.0343, *R*_sigma_ = 0.0449] |
| Data/restraints/parameters | 20225/531/1343 |
| Goodness-of-fit on F^2^ | 1.016 |
| Final R indexes [I>=2σ (I)] | *R_1_* = 0.0491, *wR_2_* = 0.1286 |
| Final R indexes [all data] | *R_1_* = 0.0595, *wR_2_* = 0.1359 |
| CCDC | 2079698 |

*R_1_ = ∑׀׀F_o_׀−׀F_c_׀׀/∑׀F_o_׀. wR_2_ = [∑w(F_o_^2^ −F_c_^2^)^2^/∑w(F_o_^2^)^2^]^1/2^*

**Table S6.** Crystal data and structure refinements of **4**.

|  | **4** |
| --- | --- |
| Empirical formula | C_111_H_92_Cu_3_F_6_OP_7_ |
| Formula weight | 1963.25 |
| Temperature/K | 200.00(10) |
| Crystal system | triclinic |
| Space group | *P*-1 |
| a/Å | 15.00700(10) |
| b/Å | 15.67330(10) |
| c/Å | 21.3373(2) |
| *α*/° | 102.5900(10) |
| *β*/° | 95.9090(10) |
| *γ*/° | 99.4000(10) |
| Volume/Å3 | 4781.99(7) |
| Z | 2 |
| ρcalcg/cm^3^ | 1.363 |
| *μ*/mm^‒1^ | 2.380 |
| F(000) | 2024.0 |
| Crystal size/mm^3^ | 0.08 × 0.07 × 0.06 |
| Radiation | CuKα (λ = 1.54184) |
| 2θ range for data collection/° | 6.882 to 148.328 |
| Index ranges | -18 ≤ h ≤ 17, -17 ≤ k ≤ 19, -26 ≤ l ≤ 26 |
| Reflections collected | 56345 |
| Independent reflections | 18798 [*R*_int_ = 0.0358, *R*_sigma_ = 0.0382] |
| Data/restraints/parameters | 18798/663/1282 |
| Goodness-of-fit on F^2^ | 1.046 |
| Final R indexes [I>=2σ (I)] | *R_1_* = 0.0697, *wR_2_* = 0.2135 |
| Final R indexes [all data] | *R_1_* = 0.0848, *wR_2_* = 0.2237 |
| CCDC | 2079699 |

*R_1_ = ∑׀׀F_o_׀−׀F_c_׀׀/∑׀F_o_׀. wR_2_ = [∑w(F_o_^2^ −F_c_^2^)^2^/∑w(F_o_^2^)^2^]^1/2^*

**Table S7.** Crystal data and structure refinements of **5**.

|  | **5** |
| --- | --- |
| Empirical formula | C_111_H_88_Cu_3_F_6_N_2_O_4_P_7_ |
| Formula weight | 2035.24 |
| Temperature/K | 297.2(5) |
| Crystal system | monoclinic |
| Space group | *P*2_1_/*c* |
| a/Å | 14.1025(7) |
| b/Å | 30.140(3) |
| c/Å | 24.3407(13) |
| *α*/° | 90 |
| *β*/° | 94.237(5) |
| *γ*/° | 90 |
| Volume/Å3 | 10317.6(12) |
| Z | 4 |
| ρcalcg/cm^3^ | 1.310 |
| *μ*/mm^‒1^ | 2.255 |
| F(000) | 4184.0 |
| Crystal size/mm^3^ | 0.08 × 0.08 × 0.07 |
| Radiation | CuKα (λ = 1.54184) |
| 2θ range for data collection/° | 6.904 to 124.998 |
| Index ranges | -16 ≤ h ≤ 12, -34 ≤ k ≤ 34, -28 ≤ l ≤ 28 |
| Reflections collected | 53292 |
| Independent reflections | 16130 [*R*_int_ = 0.1705, *R*_sigma_ = 0.2132] |
| Data/restraints/parameters | 16130/532/1268 |
| Goodness-of-fit on F^2^ | 1.088 |
| Final R indexes [I>=2σ (I)] | *R_1_* = 0.1221, *wR_2_* = 0.3067 |
| Final R indexes [all data] | *R_1_* = 0.2308, *wR_2_* = 0.3741 |
| CCDC | 2079701 |

*R_1_ = ∑׀׀F_o_׀−׀F_c_׀׀/∑׀F_o_׀. wR_2_ = [∑w(F_o_^2^ −F_c_^2^)^2^/∑w(F_o_^2^)^2^]^1/2^*

**Table S8.** Bond lengths for **1**.

| **Atom-Atom** | **Bond length (Å)** | **Atom-Atom** | **Bond length (Å)** |
| --- | --- | --- | --- |
| Cu2-Cu1 | 2.6943(8) | Cu3-P4 | 2.2693(15) |
| Cu2-Cu3 | 2.6189(9) | Cu3-C6 | 2.150(5) |
| Cu2-P2 | 2.2803(15) | Cu3-C4 | 2.086(6) |
| Cu2-P3 | 2.2939(15) | P6-C3 | 1.827(5) |
| Cu2-C6 | 2.077(6) | P5-C3 | 1.821(6) |
| Cu2-C4 | 2.405(6) | P2-C1 | 1.841(5) |
| Cu1-Cu3 | 2.5275(9) | P1-C1 | 1.846(6) |
| Cu1-P6 | 2.2783(14) | P3-C2 | 1.839(6) |
| Cu1-P1 | 2.2680(15) | P4-C2 | 1.847(6) |
| Cu1-C6 | 2.311(5) | C5-C4 | 1.234(9) |
| Cu1-C4 | 2.103(6) | C7-C6 | 1.222(8) |
| Cu3-P5 | 2.2904(15) |  |  |

**Table S9.** Bond angles for **1**.

| **Atom-Atom-Atom** | **Bond angle (˚)** | **Atom-Atom-Atom** | **Bond angle (˚)** |
| --- | --- | --- | --- |
| Cu3-Cu2-Cu1 | 56.79(2) | P5-Cu3-Cu1 | 96.42(4) |
| P2-Cu2-Cu1 | 91.86(4) | P4-Cu3-Cu2 | 96.04(4) |
| P2-Cu2-Cu3 | 148.22(4) | P4-Cu3-Cu1 | 154.12(5) |
| P2-Cu2-P3 | 115.78(6) | P4-Cu3-P5 | 108.17(6) |
| P2-Cu2-C4 | 107.91(14) | C6-Cu3-Cu2 | 50.48(15) |
| P3-Cu2-Cu1 | 149.22(5) | C6-Cu3-Cu1 | 58.54(14) |
| P3-Cu2-Cu3 | 93.87(4) | C6-Cu3-P5 | 102.42(15) |
| P3-Cu2-C4 | 106.79(15) | C6-Cu3-P4 | 121.18(15) |
| C6-Cu2-Cu1 | 56.15(14) | C4-Cu3-Cu2 | 60.23(16) |
| C6-Cu2-Cu3 | 52.99(15) | C4-Cu3-Cu1 | 53.20(16) |
| C6-Cu2-P2 | 116.32(16) | C4-Cu3-P5 | 124.96(17) |
| C6-Cu2-P3 | 115.91(16) | C4-Cu3-P4 | 104.11(17) |
| C6-Cu2-C4 | 90.0(2) | C4-Cu3-C6 | 97.2(2) |
| C4-Cu2-Cu1 | 48.30(14) | C3-P6-Cu1 | 111.65(19) |
| C4-Cu2-Cu3 | 48.84(14) | C3-P5-Cu3 | 110.70(18) |
| Cu3-Cu1-Cu2 | 60.10(2) | C1-P2-Cu2 | 110.96(19) |
| P6-Cu1-Cu2 | 148.67(4) | C1-P1-Cu1 | 112.25(19) |
| P6-Cu1-Cu3 | 95.58(4) | C2-P3-Cu2 | 110.6(2) |
| P6-Cu1-C6 | 101.95(14) | C2-P4-Cu3 | 111.7(2) |
| P1-Cu1-Cu2 | 95.34(4) | Cu2-C6-Cu1 | 75.55(18) |
| P1-Cu1-Cu3 | 151.31(5) | Cu2-C6-Cu3 | 76.53(19) |
| P1-Cu1-P6 | 112.32(5) | Cu3-C6-Cu1 | 68.92(15) |
| P1-Cu1-C6 | 123.36(14) | C7-C6-Cu2 | 139.6(4) |
| C6-Cu1-Cu2 | 48.30(14) | C7-C6-Cu1 | 125.3(4) |
| C6-Cu1-Cu3 | 52.54(14) | C7-C6-Cu3 | 140.4(5) |
| C4-Cu1-Cu2 | 58.65(16) | Cu1-C4-Cu2 | 73.05(17) |
| C4-Cu1-Cu3 | 52.59(16) | Cu3-C4-Cu2 | 70.92(16) |
| C4-Cu1-P6 | 124.09(17) | Cu3-C4-Cu1 | 74.21(19) |
| C4-Cu1-P1 | 103.29(17) | C5-C4-Cu2 | 118.8(5) |
| C4-Cu1-C6 | 92.0(2) | C5-C4-Cu1 | 139.5(5) |
| Cu1-Cu3-Cu2 | 63.11(2) | C5-C4-Cu3 | 145.5(5) |
| P5-Cu3-Cu2 | 151.34(5) | P6-Cu1-P1 | 112.32(5) |

**Table S10.** Bond lengths for **2**.

| **Atom-Atom** | **Bond length (Å)** | **Atom-Atom** | **Bond length (Å)** |
| --- | --- | --- | --- |
| Cu2-Cu1 | 2.5957(15) | Cu3-P4 | 2.274(2) |
| Cu2-Cu3 | 2.6292(16) | Cu3-C6 | 2.210(8) |
| Cu2-P2 | 2.268(2) | Cu3-C4 | 2.126(7) |
| Cu2-P3 | 2.267(2) | P6-C3 | 1.809(7) |
| Cu2-C6 | 2.078(8) | P5-C3 | 1.844(7) |
| Cu2-C4 | 2.387(8) | P2-C1 | 1.835(9) |
| Cu1-Cu3 | 2.5824(14) | P1-C1 | 1.833(8) |
| Cu1-P6 | 2.2638(19) | P3-C2 | 1.786(9) |
| Cu1-P1 | 2.284(2) | P4-C2 | 1.850(8) |
| Cu1-C6 | 2.333(8) | C5-C4 | 1.27(4) |
| Cu1-C4 | 2.061(7) | C7-C6 | 1.189(11) |
| Cu3-P5 | 2.279(2) |  |  |

**Table S11.** Bond angles for **2**.

| **Atom-Atom-Atom** | **Bond angle (˚)** | **Atom-Atom-Atom** | **Bond angle (˚)** |
| --- | --- | --- | --- |
| Cu3-Cu2-Cu1 | 59.24(4) | P5-Cu3-Cu1 | 94.98(6) |
| P2-Cu2-Cu1 | 92.19(7) | P4-Cu3-Cu2 | 95.92(6) |
| P2-Cu2-Cu3 | 151.21(7) | P4-Cu3-Cu1 | 149.78(7) |
| P2-Cu2-P3 | 113.01(9) | P4-Cu3-P5 | 113.30(8) |
| P2-Cu2-C4 | 109.78(19) | C6-Cu3-Cu2 | 50.0(2) |
| P3-Cu2-Cu1 | 149.17(8) | C6-Cu3-Cu1 | 57.6(2) |
| P3-Cu2-Cu3 | 93.42(7) | C6-Cu3-P5 | 101.8(2) |
| P3-Cu2-C4 | 103.8(2) | C6-Cu3-P4 | 122.1(2) |
| C6-Cu2-Cu1 | 58.6(2) | C4-Cu3-Cu2 | 59.1(2) |
| C6-Cu2-Cu3 | 54.5(2) | C4-Cu3-Cu1 | 50.8(2) |
| C6-Cu2-P2 | 115.8(3) | C4-Cu3-P5 | 121.2(2) |
| C6-Cu2-P3 | 119.1(3) | C4-Cu3-P4 | 102.7(2) |
| C6-Cu2-C4 | 92.0(3) | C4-Cu3-C6 | 95.9(3) |
| C4-Cu2-Cu1 | 48.64(18) | C3-P6-Cu1 | 111.9(2) |
| C4-Cu2-Cu3 | 49.86(18) | C3-P5-Cu3 | 111.3(2) |
| Cu3-Cu1-Cu2 | 61.03(4) | C1-P2-Cu2 | 107.5(3) |
| P6-Cu1-Cu2 | 150.07(7) | C1-P1-Cu1 | 109.6(3) |
| P6-Cu1-Cu3 | 95.45(6) | C2-P3-Cu2 | 112.4(3) |
| P6-Cu1-C6 | 102.26(19) | C2-P4-Cu3 | 110.4(3) |
| P1-Cu1-Cu2 | 95.94(7) | Cu2-C6-Cu1 | 71.8(2) |
| P1-Cu1-Cu3 | 151.53(7) | Cu2-C6-Cu3 | 75.6(3) |
| P1-Cu1-P6 | 111.28(8) | Cu3-C6-Cu1 | 69.2(2) |
| P1-Cu1-C6 | 125.7(2) | C7-C6-Cu2 | 144.2(7) |
| C6-Cu1-Cu2 | 49.53(19) | C7-C6-Cu1 | 133.7(7) |
| C6-Cu1-Cu3 | 53.1(2) | C7-C6-Cu3 | 132.1(7) |
| C4-Cu1-Cu2 | 60.4(2) | Cu1-C4-Cu2 | 71.0(2) |
| C4-Cu1-Cu3 | 53.1(2) | Cu3-C4-Cu2 | 71.0(2) |
| C4-Cu1-P6 | 122.0(2) | Cu3-C4-Cu1 | 76.2(2) |
| C4-Cu1-P1 | 102.2(2) | C5-C4-Cu2 | 142.2(19) |
| C4-Cu1-C6 | 94.0(3) | C5-C4-Cu1 | 140(2) |
| Cu1-Cu3-Cu2 | 59.74(4) | C5-C4-Cu3 | 127.7(19) |
| P5-Cu3-Cu2 | 148.50(7) | P6-Cu1-P1 | 111.28(8) |

**Table S12.** Bond lengths for **3**.

| **Atom-Atom** | **Bond length (Å)** | **Atom-Atom** | **Bond length (Å)** |
| --- | --- | --- | --- |
| Cu2-Cu1 | 2.4642(5) | Cu3-P4 | 2.2923(7) |
| Cu2-Cu3 | 2.6644(5) | Cu3-C4 | 2.067(2) |
| Cu2-P2 | 2.3265(7) | P6-C3 | 1.820(3) |
| Cu2-P3 | 2.2575(7) | P5-C3 | 1.845(3) |
| Cu2-C6 | 2.058(3) | P2-C1 | 1.832(3) |
| Cu2-C4 | 2.111(3) | P1-C1 | 1.842(3) |
| Cu1-P6 | 2.2293(7) | P3-C2 | 1.836(3) |
| Cu1-P1 | 2.2608(7) | P4-C2 | 1.844(3) |
| Cu1-C6 | 2.096(3) | C5-C4 | 1.219(4) |
| Cu1-C4 | 2.257(2) | C7-C6 | 1.201(4) |
| Cu3-P5 | 2.2617(7) |  |  |

**Table S13.** Bond angles for **3**.

| **Atom-Atom-Atom** | **Bond angle (˚)** | **Atom-Atom-Atom** | **Bond angle (˚)** |
| --- | --- | --- | --- |
| Cu3-Cu2-Cu1 | 78.916(15) | C4-Cu1-C6 | 100.52(10) |
| P2-Cu2-Cu1 | 96.40(2) | P5-Cu3-Cu2 | 120.24(2) |
| P2-Cu2-Cu3 | 147.46(2) | P4-Cu3-Cu2 | 98.66(2) |
| P2-Cu2-P3 | 105.48(3) | P4-Cu3-P5 | 134.32(3) |
| P2-Cu2-C4 | 100.34(7) | C4-Cu3-Cu2 | 51.12(7) |
| P3-Cu2-Cu1 | 158.10(2) | C4-Cu3-P5 | 119.33(7) |
| P3-Cu2-Cu3 | 81.96(2) | C4-Cu3-P4 | 103.15(7) |
| P3-Cu2-C4 | 115.03(7) | C3-P6-Cu1 | 108.59(9) |
| C6-Cu2-Cu1 | 54.32(7) | C3-P5-Cu3 | 119.32(8) |
| C6-Cu2-Cu3 | 91.46(7) | C1-P2-Cu2 | 106.86(9) |
| C6-Cu2-P2 | 112.09(7) | C1-P1-Cu1 | 107.97(9) |
| C6-Cu2-P3 | 116.04(8) | C2-P3-Cu2 | 109.32(9) |
| C6-Cu2-C4 | 106.83(10) | C2-P4-Cu3 | 106.53(8) |
| C4-Cu2-Cu1 | 58.50(7) | Cu2-C6-Cu1 | 72.76(8) |
| C4-Cu2-Cu3 | 49.65(7) | C7-C6-Cu2 | 145.3(2) |
| P6-Cu1-Cu2 | 137.65(2) | C7-C6-Cu1 | 141.9(2) |
| P6-Cu1-C6 | 102.65(8) | Cu1-C4-Cu2 | 68.59(7) |
| P1-Cu1-Cu2 | 98.14(2) | Cu3-C4-Cu2 | 79.23(9) |
| P1-Cu1-P6 | 124.20(3) | Cu3-C4-Cu1 | 97.89(10) |
| P1-Cu1-C6 | 113.65(7) | C5-C4-Cu2 | 159.6(2) |
| C6-Cu1-Cu2 | 52.92(7) | C5-C4-Cu1 | 123.5(2) |
| C4-Cu1-Cu2 | 52.91(7) | C5-C4-Cu3 | 112.34(19) |
| C4-Cu1-P6 | 112.94(7) | P6-Cu1-P1 | 124.20(3) |
| C4-Cu1-P1 | 100.71(6) |  |  |

**Table S14.** Bond lengths for **4**.

| **Atom-Atom** | **Bond length (Å)** | **Atom-Atom** | **Bond length (Å)** |
| --- | --- | --- | --- |
| Cu2-Cu1 | 2.6907(8) | Cu3-P4 | 2.2703(11) |
| Cu2-Cu3 | 2.7452(8) | Cu3-C6 | 2.361(4) |
| Cu2-P2 | 2.3142(13) | Cu3-C4 | 2.126(4) |
| Cu2-P3 | 2.2976(11) | P6-C3 | 1.843(4) |
| Cu2-C6 | 2.131(4) | P5-C3 | 1.831(4) |
| Cu2-C4 | 2.367(4) | P2-C1 | 1.822(5) |
| Cu1-Cu3 | 2.5724(8) | P1-C1 | 1.828(5) |
| Cu1-P6 | 2.2980(10) | P3-C2 | 1.893(4) |
| Cu1-P1 | 2.2667(11) | P4-C2 | 1.840(4) |
| Cu1-C6 | 2.180(4) | C5-C4 | 1.155(6) |
| Cu1-C4 | 2.162(4) | C7-C6 | 1.172(6) |
| Cu3-P5 | 2.2812(10) |  |  |

**Table S15.** Bond angles for **4**.

| **Atom-Atom-Atom** | **Bond angle (˚)** | **Atom-Atom-Atom** | **Bond angle (˚)** |
| --- | --- | --- | --- |
| Cu3-Cu2-Cu1 | 56.477(19) | P5-Cu3-Cu1 | 93.64(3) |
| P2-Cu2-Cu1 | 93.60(4) | P4-Cu3-Cu2 | 95.37(3) |
| P2-Cu2-Cu3 | 146.93(4) | P4-Cu3-Cu1 | 153.52(4) |
| P2-Cu2-P3 | 118.43(5) | P4-Cu3-P5 | 111.83(4) |
| P2-Cu2-C4 | 102.90(11) | C6-Cu3-Cu2 | 48.61(10) |
| P3-Cu2-Cu1 | 147.10(4) | C6-Cu3-Cu1 | 52.24(10) |
| P3-Cu2-Cu3 | 90.64(3) | C6-Cu3-P5 | 107.64(10) |
| P3-Cu2-C4 | 110.62(11) | C6-Cu3-P4 | 121.55(9) |
| C6-Cu2-Cu1 | 52.20(10) | C4-Cu3-Cu2 | 56.44(11) |
| C6-Cu2-Cu3 | 56.23(10) | C4-Cu3-Cu1 | 53.79(11) |
| C6-Cu2-P2 | 118.91(11) | C4-Cu3-P5 | 119.25(12) |
| C6-Cu2-P3 | 110.87(11) | C4-Cu3-P4 | 104.77(12) |
| C6-Cu2-C4 | 90.65(15) | C4-Cu3-C6 | 90.95(15) |
| C4-Cu2-Cu1 | 50.12(10) | C3-P6-Cu1 | 109.87(12) |
| C4-Cu2-Cu3 | 48.44(11) | C3-P5-Cu3 | 110.33(13) |
| Cu3-Cu1-Cu2 | 62.83(2) | C1-P2-Cu2 | 110.26(15) |
| P6-Cu1-Cu2 | 152.43(4) | C1-P1-Cu1 | 112.55(17) |
| P6-Cu1-Cu3 | 96.62(3) | C2-P3-Cu2 | 109.73(15) |
| P6-Cu1-C6 | 103.90(11) | C2-P4-Cu3 | 111.65(15) |
| P1-Cu1-Cu2 | 94.30(3) | Cu2-C6-Cu1 | 77.24(14) |
| P1-Cu1-Cu3 | 150.98(4) | Cu2-C6-Cu3 | 75.16(12) |
| P1-Cu1-P6 | 110.26(4) | Cu3-C6-Cu1 | 68.88(11) |
| P1-Cu1-C6 | 121.45(11) | C7-C6-Cu2 | 146.3(3) |
| C6-Cu1-Cu2 | 50.56(10) | C7-C6-Cu1 | 135.5(3) |
| C6-Cu1-Cu3 | 58.88(10) | C7-C6-Cu3 | 119.3(3) |
| C4-Cu1-Cu2 | 57.15(11) | Cu1-C4-Cu2 | 72.73(13) |
| C4-Cu1-Cu3 | 52.49(12) | Cu3-C4-Cu2 | 75.11(14) |
| C4-Cu1-P6 | 126.21(11) | Cu3-C4-Cu1 | 73.72(14) |
| C4-Cu1-P1 | 100.88(12) | C5-C4-Cu2 | 111.6(3) |
| C4-Cu1-C6 | 95.05(15) | C5-C4-Cu1 | 140.7(4) |
| Cu1-Cu3-Cu2 | 60.69(2) | C5-C4-Cu3 | 145.5(4) |
| P5-Cu3-Cu2 | 151.90(3) | P6-Cu1-P1 | 110.26(4) |

**Table S16.** Bond lengths for **5**.

| **Atom-Atom** | **Bond length (Å)** | **Atom-Atom** | **Bond length (Å)** |
| --- | --- | --- | --- |
| Cu2-Cu1 | 2.690(2) | Cu3-P4 | 2.290(4) |
| Cu2-Cu3 | 2.6199(19) | Cu3-C6 | 2.155(11) |
| Cu2-P2 | 2.272(3) | Cu3-C4 | 2.163(14) |
| Cu2-P3 | 2.289(3) | P6-C3 | 1.809(12) |
| Cu2-C6 | 2.006(12) | P5-C3 | 1.825(12) |
| Cu2-C4 | 2.366(13) | P2-C1 | 1.847(10) |
| Cu1-Cu3 | 2.788(2) | P1-C1 | 1.829(10) |
| Cu1-P6 | 2.247(3) | P3-C2 | 1.835(10) |
| Cu1-P1 | 2.292(3) | P4-C2 | 1.815(11) |
| Cu1-C4 | 1.996(14) | C5-C4 | 1.149(17) |
| Cu3-P5 | 2.282(3) | C7-C6 | 1.107(16) |

**Table S17.** Bond angles for **5**.

| **Atom-Atom-Atom** | **Bond angle (˚)** | **Atom-Atom-Atom** | **Bond angle (˚)** |
| --- | --- | --- | --- |
| Cu3-Cu2-Cu1 | 63.33(6) | P4-Cu3-Cu2 | 95.38(9) |
| P2-Cu2-Cu1 | 86.57(9) | P4-Cu3-Cu1 | 139.95(11) |
| P2-Cu2-Cu3 | 149.89(11) | P4-Cu3-P5 | 120.43(12) |
| P2-Cu2-P3 | 113.21(11) | C6-Cu3-Cu2 | 48.5(3) |
| P2-Cu2-C4 | 108.6(3) | C6-Cu3-Cu1 | 70.8(3) |
| P3-Cu2-Cu1 | 149.00(10) | C6-Cu3-P5 | 99.0(3) |
| P3-Cu2-Cu3 | 94.70(9) | C6-Cu3-P4 | 117.7(3) |
| P3-Cu2-C4 | 103.5(3) | C4-Cu3-Cu2 | 58.4(4) |
| C6-Cu2-Cu1 | 75.0(3) | C4-Cu3-Cu1 | 45.4(4) |
| C6-Cu2-Cu3 | 53.6(3) | C4-Cu3-P5 | 124.0(4) |
| C6-Cu2-P2 | 120.6(3) | C4-Cu3-P4 | 95.6(4) |
| C6-Cu2-P3 | 110.7(3) | C4-Cu3-C6 | 99.7(5) |
| C6-Cu2-C4 | 97.7(5) | C3-P6-Cu1 | 114.4(4) |
| C4-Cu2-Cu1 | 46.0(3) | C3-P5-Cu3 | 109.8(4) |
| C4-Cu2-Cu3 | 51.1(4) | C1-P2-Cu2 | 110.0(4) |
| Cu3-Cu1-Cu2 | 57.12(5) | C1-P1-Cu1 | 108.6(4) |
| P6-Cu1-Cu2 | 137.01(11) | C2-P3-Cu2 | 107.8(4) |
| P6-Cu1-Cu3 | 91.29(10) | C2-P4-Cu3 | 110.8(4) |
| P1-Cu1-Cu2 | 97.73(9) | Cu2-C6-Cu3 | 77.9(4) |
| P1-Cu1-Cu3 | 149.63(11) | C7-C6-Cu2 | 151.7(11) |
| P1-Cu1-P6 | 118.83(13) | C7-C6-Cu3 | 125.3(10) |
| C4-Cu1-Cu2 | 58.4(4) | Cu1-C4-Cu2 | 75.6(4) |
| C4-Cu1-Cu3 | 50.5(4) | Cu3-C4-Cu2 | 70.5(4) |
| C4-Cu1-P6 | 125.4(4) | Cu3-C4-Cu1 | 84.1(6) |
| C4-Cu1-P1 | 103.4(4) | C5-C4-Cu2 | 141.0(13) |
| Cu1-Cu3-Cu2 | 59.56(5) | C5-C4-Cu1 | 132.4(12) |
| P5-Cu3-Cu2 | 141.84(11) | C5-C4-Cu3 | 129.2(11) |
| P5-Cu3-Cu1 | 94.29(10) | P6-Cu1-P1 | 118.83(13) |
